# Identifying risk factors for the prognosis of head and neck cutaneous squamous cell carcinoma: A systematic review and meta-analysis

**DOI:** 10.1371/journal.pone.0239586

**Published:** 2020-09-29

**Authors:** Shihua Zeng, Lixin Fu, Peimei Zhou, Hua Ling

**Affiliations:** 1 Department of Dermatology, Chengdu Second People’s Hospital, Chengdu, China; 2 Department of Anesthesiology, Chengdu Second People’s Hospital, Chengdu, China; Emory University Hospital Midtown, UNITED STATES

## Abstract

In this study, we sought to identify the potential impacts of disease characteristics on the prognosis of cutaneous squamous cell carcinoma (cSCC). We searched the PubMed, EmBase, and Cochrane Library databases from their inception until February 2020 to identify studies that investigated the prognosis of cSCC. The pooled effect estimates were applied using odds ratios (OR) and 95% confidence intervals (CI) and were calculated using the random-effects model. Forty-three studies including a total of 21,530 patients and reporting 28,627 cases of cSCC were selected for the final meta-analysis. Poor differentiation (OR, 3.54; 95% CI, 2.30–5.46; *P* < 0.001), perineural invasion (OR, 3.27; 95% CI, 1.60–6.67; *P* = 0.001), Breslow greater than 2 mm (OR, 5.47; 95% CI, 2.63–11.37; *P* < 0.001), diameter greater than 20 mm (OR, 4.62; 95% CI, 2.95–7.23; *P* < 0.001), and location on temple (OR, 3.20; 95% CI, 1.12–9.15; *P* = 0.030) were associated with an increased risk of recurrence, whereas immunosuppression status and location on cheek, ear, or lip were not associated with the risk of recurrence. Poor differentiation (OR, 6.82; 95% CI, 4.66–9.99; *P* < 0.001); perineural invasion (OR, 7.15; 95% CI, 4.73–10.83; *P* < 0.001); Breslow greater than 2 mm (OR, 6.11; 95% CI, 4.05–9.21; *P* < 0.001); diameter greater than 20 mm (OR, 5.01; 95% CI, 2.56–9.80; *P* < 0.001); and location on ear (OR, 2.38; 95% CI, 1.39–4.09; *P* = 0.002), lip (OR, 2.15; 95% CI, 1.26–3.68; *P* = 0.005), and temple (OR, 2.77; 95% CI, 1.20–6.40; *P* = 0.017) were associated with an increased risk of metastasis, whereas immunosuppression status and location on cheek did not affect the risk of metastasis. Finally, poor differentiation (OR, 5.97; 95% CI, 1.82–19.62; *P* = 0.003), perineural invasion (OR, 6.64; 95% CI, 3.63–12.12; *P* < 0.001), and Breslow greater than 2 mm (OR, 3.42; 95% CI, 1.76–6.66; *P* < 0.001) were associated with an increased risk of disease-specific death, whereas diameter; immunosuppression status; and location on ear, lip, and temple did not affect the risk of disease-specific death. We found that differentiation, perineural invasion, depth, diameter, and location could affect the prognosis of cSCC. The potential role of other patient characteristics on the prognosis of cSCC should be identified in further large-scale prospective studies.

## Introduction

Nonmelanoma skin cancer is the most common type of cancer, and the number of cases has increased to more than 1 million in the United States annually [1–3]. Cutaneous squamous cell carcinoma (cSCC) accounts for 20%–30% of nonmelanoma skin cancer, and most patients present with a head or neck location [4]. Most cases of cSCC can now be cured through surgery, but a certain subset of patients with high-risk cSCC could experience local recurrence, nodal metastasis, and disease-specific death (DSD) [5]. Although the prevalence of local recurrence, nodal metastasis, and DSD was low, the prognosis of these cases was poor, and the 5-year survival rate was only 30% to 60% for patients treated surgically for lymph node metastases from cSCC of head and neck [6].

The incidence of head and neck cSCC is increasing, and the specific anatomic subsites have been associated with an increased risk of regional metastatic involvement [7]. The parotid gland has been identified as a common location for spread because it could receive lymphatics from the ear, forehead, face, temple, and scalp [8]. Cancers with involvement of the parotid gland frequently demonstrated macroscopic or microscopic cervical metastases [9]. Other clinicopathological features, including differentiation, perineural invasion, depth, diameter, and immunosuppression, might also affect the prognosis of cSCC [10]. However, the predictive value of these clinicopathological features on the prognosis of cSCC vary. Therefore, the current systematic review and meta-analysis was conducted based on all available evidence to obtain pooled results regarding the predictive value of clinicopathological characteristics on the prognosis of cSCC.

## Materials and methods

### Data sources, search strategy, and selection criteria

This study was performed and reported following the Preferred Reporting Items for Systematic Reviews and Meta-Analysis Statement [11]. Studies with an observational design that investigated the role of clinicopathological characteristics on the prognosis of cSCC were eligible for inclusion in this study, and restrictions were not applied for publication language or status (published, in press, or in progress). The electronic databases of PubMed, EmBase, and the Cochrane Library were systematically searched from their inception until February 2020 using the following search terms: (skin or cutaneous or dermal or cutanea) AND (squamous or epidermoid or planocellular or “prickle cell” or verrucous) AND (carcinoma*) AND (outcome* or recurrence* or relaps* or recrudescence* or recurrent or recidive or metastas* or metastatic* or spread* or disseminat* or secondary or migrat* or death or morbidity or mortality or surviv*) AND (risk or risks or “perineural invasion*” or “peri-neural invasion*” or PNI or depth or thickness or size or diameter or or location* or ear or ears or cheek* or lip or lips or differentiation or immunocompromised or “immune compromi*” or “sentinel lymph node*”). Details regarding the search strategy are presented in S1 Appendix. The reference lists of retrieved studies were also reviewed manually to identify any additional studies that met the inclusion criteria.

Two reviewers independently conducted the literature search and study selection. Any disagreements were resolved by discussion until a consensus was reached. A study was included if it met all of inclusion criteria: (1) all patients had cSCC; (2) the study reported at least 1 of the following: differentiation, perineural invasion, depth, diameter, immunosuppression status, and location; and (3) the study reported at least 1 of the following: recurrence, metastasis, and DSD. Furthermore, there were no restrictions placed on study design.

### Data collection and quality assessment

The information of included studies were collected by 2 reviewers independently. The abstracted items included the first author’s name, publication year, study design, country, sample size, mean age, number of cSCCs, outcomes, risk factors, inclusion criteria, duration of follow-up, and investigated outcomes. The quality of included studies was assessed using the Newcastle-Ottawa Scale (NOS) according to selection (4 items, 4 stars), comparability (1 item, 2 stars), and outcome (3 items, 3 stars), and the “star system” for each individual study ranged from 0 to 9 [12]. The quality assessment was conducted by 2 reviewers, and any inconsistencies were resolved by an additional reviewer who referred to the full-text of the article.

### Statistical analysis

The effect estimates for the role of clinicopathological characteristics on the prognosis of cSCC were assigned as odds ratios (OR) and 95% confidence intervals (CI), because the analysis included both prospective and retrospective studies. The random-effects model was applied to calculate the pooled effect estimates because it could consider the underlying variations across the included studies [13, 14]. *I*^*2*^ and the Q statistic were applied to assess the heterogeneity among the included studies. Significant heterogeneity was defined as *I*^*2*^ greater than 50.0% or a *P* value less than 0.10 [15, 16]. Sensitivity analysis was conducted for outcomes that reported 5 or more studies to assess the robustness of the pooled conclusions by sequential exclusion of individual studies [17]. Publication bias for outcomes that reported 5 or more studies was assessed using funnel plots, and the Egger and Begg tests [18, 19]. *P* values of less than 0.10 were considered as potential publication bias for the Egger and Begg tests. The inspection level for pooled results was 2-sided, and a *P* value less than 0.05 was regarded as statistically significant. All statistical analyses in this study were conducted using the STATA software (version 10.0; StataCorp, College Station, TX).

## Results

### Literature search

**Fig 1:**
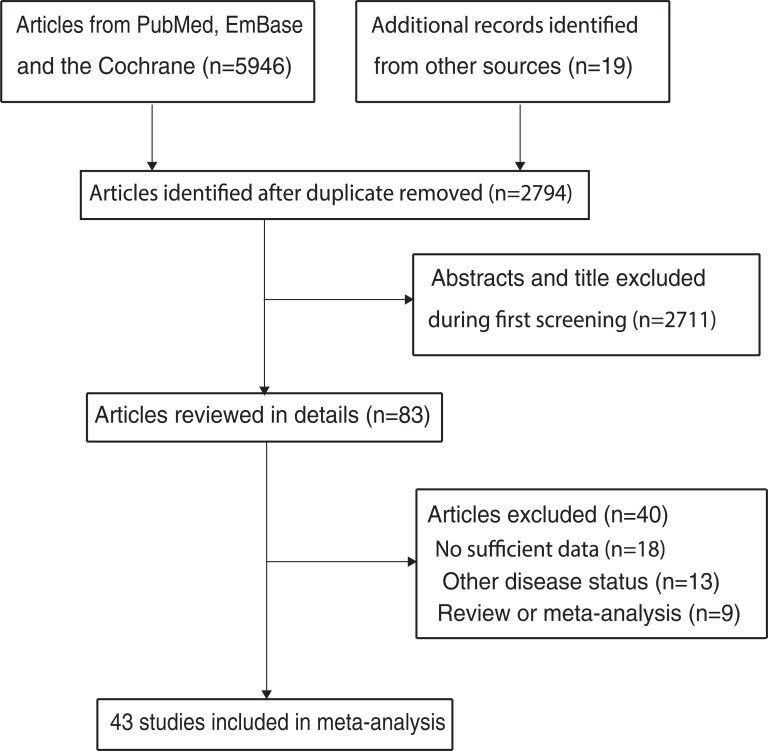
Flow diagram of the literature search and study selection process.

The electronic searches in PubMed, EmBase, and the Cochrane Library yielded 5946 articles, 2794 of which were retained after duplicate records were excluded. Of the remaining articles, 2711 were excluded because they reported irrelevant titles. The remaining 83 studies were retrieved for further full-text evaluation. Forty more studies were excluded for the following reasons: insufficient data (n = 18), other disease status (n = 13), and review or meta-analysis (n = 9). A total of 43 studies were selected for the final meta-analysis (Fig 1) [20–62]. Nineteen relevant studies were identified through reviewing the reference lists of the included studies. However, these studies were excluded as duplicate records because they had been included in the initial electronic searches.

### Study characteristics

Table 1 summarizes the baseline characteristics of the included studies and patients. A total of 21,530 patients and 28,627 cSCCs were included. Seven studies were designed as prospective, and the remaining 36 studies were retrospective. Twenty-five studies were conducted in Europe, 17 studies were conducted in America, and 1 study was conducted in Asia. The sample size ranged from 41 to 6164, and the duration of follow-up ranged from 1.0 to 10.0 years. The quality of the studies was assessed using the NOS, and the number of NOS stars ranged from 5 to 9, with the median of 8 stars (interquartile range, 7–9 stars).

**Table 1:** Baseline characteristics of included studies and patients.

Study	Study design	Country	Sample size	Mean age (years)	No. Of cSCC	Outcomes	Risk factors	Inclusion criteria	Follow-up duration	NOS score
Immerman 1983 [20]	Retrospective	US	86	NA	86	Recurrence	Differentiation	Biopsy-proven cSCC	4.5 years	9
Goepfert 1984 [21]	Retrospective	US	520	64.0	967	Recurrence, metastasis, disease-specific death	PNI	Biopsy-proven cSCC	2.0 years	8
Friedman 1985 [22]	Retrospective	US	63	NA	71	Recurrence, disease-specific death	Depth, differentiation	Biopsy-proven cSCC	8.0–18.0 years	9
Dinehart 1989 [23]	Retrospective	US	365	67.2	365	Metastasis	Location	Biopsy-proven cSCC; treated with Mohs	1.7 years	7
Breuninger 1990 [24]	Retrospective	Germany	571	NA	673	Metastasis	Location, diameter, differentiation, depth	Biopsy-proven cSCC	6.0 years	8
Stein 1994 [25]	Retrospective	US	44	63.5	44	Metastasis	Differentiation, depth	Biopsy-proven cSCC of lip	4.4 years	8
Pereira 1994 [26]	Retrospective	Portugal	43	NA	43	Recurrence	Diameter	Biopsy-proven cSCC; treated with ED&C followed by cryotherapy	4.0 years	5
Eroğlu 1996 [27]	Retrospective	Turkey	1,039	60.6	1,039	Recurrence	Location, diameter, differentiation	Biopsy-proven cSCC	2.3 years	8
Baker 2001 [28]	Retrospective	UK	183	78.0	227	Regional metastasis	Location	Biopsy-proven cSCC	5.0 years	8
Griffiths 2002 [29]	Retrospective	UK	157	73.7	157	Metastasis, disease-specific death	Location	Biopsy-proven cSCC	5.0 years	9
Cherpelis 2002 [30]	Retrospective	US	200	NA	200	Metastasis	Location, diameter, differentiation, PNI	Biopsy-proven cSCC; treated with Mohs	0.5–10.0 years	8
Faustina 2004 [31]	Retrospective	US	111	64.0	111	Metastasis	PNI	Biopsy-proven cSCC; periocular only	6.4 years	6
Mehrany 2005 [32]	Retrospective	US	142	73.0	171	Recurrence	Differentiation, immunosuppression	Biopsy-proven cSCC; treated with Mohs	3.3 years	8
Moore 2005 [33]	Prospective	US	193	68.0	193	Metastasis	Location, differentiation, depth, PNI	Biopsy-proven head/neck cSCC	1.7 years	6
Clayman 2005 [34]	Prospective	US	210	67.1	277	Disease-specific death	Depth, PNI	Biopsy-proven cSCC	1.8 years	8
Leibovitch 2005 [35]	Prospective	Australia	1,177	64.0	1,177	Recurrence	PNI	Biopsy-proven cSCC; treated with Mohs	5.0 years	6
Quaedvlieg 2006 [36]	Retrospective	Netherlands	580	81.1	915	Metastasis	Differentiation, PNI, depth	Biopsy-proven cSCC	5.7 years	7
Mullen 2006 [37]	Retrospective	US	136	67.0	149	Recurrence	Diameter, differentiation	Biopsy-proven cSCC	2.4 years	9
Harwood 2006 [38]	Retrospective	UK	65	68.4	100	Recurrence, metastasis	Immunosuppression	Biopsy-proven primary cSCC; Immunocompromised group (OTR) with immunocompetent control group	10.0 years	7
Brantsch 2008 [39]	Prospective	Germany	615	73.0	615	Recurrence, metastasis	Depth, diameter, location, differentiation, immunosuppression	Biopsy-proven cSCC	3.6 years	9
Mourouzis 2009 [40]	Retrospective	UK	194	62.0–104.0	218	Metastasis	Location, differentiation	Biopsy-proven cSCC of the head and neck; treated with excision	3.0 years	8
Dormand 2010 [41]	Retrospective	UK	243	77.0	517	Metastasis	Differentiation	Biopsy-proven cSCC; extremities only	6.2 years	7
Pugliano-Mauro 2010 [42]	Retrospective	US	215	70.6	260	Metastasis	Location	Biopsy-proven high risk cSCC,including recurrent tumors; treated with Mohs	3.9 years	7
Kyrgidis 2010 [43]	Prospective	Greece	315	71.9	315	Disease-specific death	Depth, differentiation, PNI	Biopsy-proven cSCC	3.9 years	9
Brougham 2012 [44]	Retrospective	New Zealand	6,164	74.0	8,997	Metastasis	Location, PNI, differentiation	Biopsy-proven cSCC	5.9 years	8
Metchnikoff 2012 [45]	Retrospective	US	41	53.3	225	Recurrence	Diameter, differentiation, PNI	Biopsy-proven cSCC in heart/lung transplant recipients	1.3 years	8
Peat 2012 [46]	Retrospective	New Zealand	170	76.0	170	Metastasis	Differentiation, PNI	Biopsy-proven cSCC	5.0 years	9
Toll 2012 [47]	Retrospective	Spain	101	77.7	101	Metastasis	Differentiation, PNI	iopsy-proven cSCC; metastatic group vs Nonmetastatic control group	2.0 years	9
Schmults 2013 [48]	Retrospective	US	985	71.0	1,832	Recurrence, metastasis, disease-specific death	Location, PNI	Biopsy-proven cSCC; excluded SCCIS, recurrent SCC	4.2 years	9
Roozeboom 2013 [49]	Retrospective	Netherlands	224	72.0	224	Recurrence, metastasis	Location, depth, differentiation, PNI	Biopsy-proven cSCC	3.6 years	8
Karia 2014 [50]	Retrospective	US	974	71.0	1,818	Recurrence, metastasis, disease-specific death	Location, diameter, depth, differentiation, immunosuppression	Biopsy-proven cSCC; excluded SCCIS, recurrent SCC, eyelid/ anogenital SCC	4.2 years	9
Vasconcelos 2014 [51]	Retrospective	Brazil	61	67.1	79	Recurrence	Differentiation, PNI	Biopsy-proven cSCCof head/neck; treated surgically	5.0 years	9
Gonzalez 2014 [52]	Retrospective	Argentina	434	74.0	434	Metastasis	Differentiation	Biopsy-proven cSCC; treated with Mohs	4.7 years	7
Brinkman 2015 [53]	Retrospective	Netherlands	131	73.0	155	Metastasis, disease-specific death	Differentiation	Biopsy-proven cSCC;treated with surgical excision	6.8 years	8
Krediet 2015 [54]	Retrospective	Germany	143	73.0	143	Metastasis	Diameter, differentiation, immunosuppression	Biopsy-proven cSCC treated with excision	2.0 years	8
Wermker 2015 [55]	Retrospective	Germany	353	78.4	353	Metastasis	PNI, immunosuppression	Biopsy-proven cSCC of external ear; treated surgically	3.6 years	8
Manyam 2015 [56]	Retrospective	US	59	72.0	59	Recurrence	Differentiation, PNI, immunosuppression	Biopsy-proven cSCC treated with surgery and radiation	1.5 years	7
Schmidt 2015 [57]	Retrospective	Australia	113	74.0	113	Disease-specific death	Diameter, immunosuppression	Biopsy-proven cSCC	5.0 years	8
Hasegawa 2015 [58]	Retrospective	Japan	451	65.9	451	Metastasis	Differentiation	Biopsy-proven cSCC	5.0 years	9
Haisma 2016 [59]	Retrospective	Netherlands	336	73.0	545	Metastasis	Location, diameter, depth, differentiation, PNI, immunosuppression	Biopsy-proven cSCC	3.6 years	8
Eigentler 2017 [60]	Prospective	Germany	1,434	78.0	2,149	Disease-specific death	Location, diameter, depth, differentiation, immunosuppression	Biopsy-proven cSCC	3.0 years	8
Genders 2018 [61]	Retrospective	Netherlands	593	NA	593	Metastasis	Diameter, depth, differentiation	Biopsy-proven cSCC	4.0 years	7
Pyne 2019 [62]	Prospective	Australia	1,296	71.5	1,296	Recurrence	Differentiation	Biopsy-proven cSCC	9.0 years	9

### Recurrence

**Fig 2:**
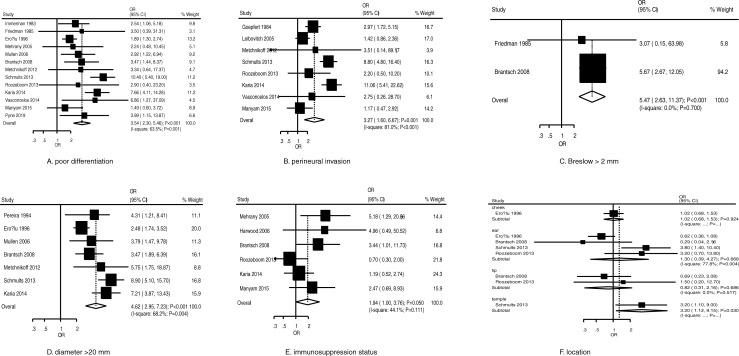
Risk factors for recurrence in patients with cSCC.

The summary results for the role of clinicopathological characteristics on the risk of recurrence for patients with cSCC are shown in Fig 2. The characteristics of poor differentiation (OR, 3.54; 95% CI, 2.30–5.46; *P* < 0.001) (Fig 2A), perineural invasion (OR, 3.27; 95% CI, 1.60–6.67; *P* = 0.001) (Fig 2B), Breslow greater than 2 mm (OR, 5.47; 95% CI, 2.63–11.37; *P* < 0.001) (Fig 2C), diameter greater than 20 mm (OR, 4.62; 95% CI, 2.95–7.23; *P* < 0.001) (Fig 2D), and location on temple (OR, 3.20; 95% CI, 1.12–9.15; *P* = 0.030) (Fig 2F) were associated with an increased risk of recurrence. However, immunosuppression status (OR, 1.94; 95% CI, 1.00–3.76; *P* = 0.050) (Fig 2E) and location on cheek (OR, 1.02; 95% CI, 0.68–1.53; *P* = 0.924) (Fig 2F), ear (OR, 1.30; 95% CI, 0.39–4.27; *P* = 0.668) (Fig 2F), or lip (OR, 0.82; 95% CI, 0.31–2.16; *P* = 0.686) (Fig 2F) did not affect the risk of recurrence in patients with cSCC. We noted significant heterogeneity for poor differentiation (*I*^*2*^ = 63.5%; *P* = 0.001), perineural invasion (*I*^*2*^ = 81.0%; *P* < 0.001), diameter greater than 20 mm (*I*^*2*^ = 68.2%; *P* = 0.004), and location on ear (*I*^*2*^ = 77.8%; *P* = 0.004). No significant heterogeneity was detected for Breslow greater than 2 mm (*I*^*2*^ = 0.0%; *P* = 0.700), immunosuppression status (*I*^*2*^ = 44.1%; *P* = 0.111), and location on lip (*I*^*2*^ = 0.0%; *P* = 0.517). Sensitivity analyses indicated that the pooled conclusions for poor differentiation, perineural invasion, diameter greater than 20 mm, and immunosuppression status were not altered by sequential exclusion of each individual study (S2 Appendix). Finally, there was no significant publication bias for the role of poor differentiation (Egger: *P* = 0.585; Begg: *P* = 0.669), perineural invasion (Egger: *P* = 0.971; Begg: *P* = 0.386), and diameter greater than 20 mm (Egger: *P* = 0.279; Begg: *P* = 0.548) on the risk of recurrence. However, we noted a potential significant publication bias for the role of immunosuppression status on the risk of recurrence (Egger: *P* = 0.082; Begg: *P* = 0.260) (S3 Appendix). However, the conclusion was not changed after adjustment for potential publication bias using the trim and fill method [63].

### Metastasis

**Fig 3:**
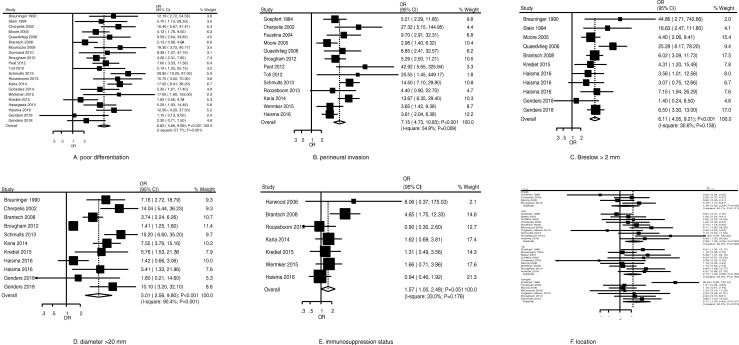
Risk factors for metastasis in patients with cSCC.

The summary results for the role of clinicopathological characteristics on the risk of metastasis for patients with cSCC are shown in Fig 3. We noted poor differentiation (OR, 6.82; 95% CI, 4.66–9.99; *P* < 0.001) (Fig 3A), perineural invasion (OR, 7.15; 95% CI, 4.73–10.83; *P* < 0.001) (Fig 3B), Breslow greater than 2 mm (OR, 6.11; 95% CI, 4.05–9.21; *P* < 0.001) (Fig 3C), diameter greater than 20 mm (OR, 5.01; 95% CI, 2.56–9.80; *P* < 0.001) (Fig 3D), location on ear (OR, 2.38; 95% CI, 1.39–4.09; *P* = 0.002) (Fig 3F), location on lip (OR, 2.15; 95% CI, 1.26–3.68; *P* = 0.005) (Fig 3F), and location on temple (OR, 2.77; 95% CI, 1.20–6.40; *P* = 0.017) (Fig 3F) were associated with an increased risk of metastasis. Immunosuppression status (OR, 1.57; 95% CI, 1.00–2.48; *P* = 0.051) (Fig 3E) and location on cheek (OR, 1.40; 95% CI, 0.60–3.29; *P* = 0.435) (Fig 3F) were not associated with the risk of metastasis. Moreover, there was potential significant heterogeneity for poor differentiation (*I*^*2*^ = 57.7%; *P* = 0.001); perineural invasion (*I*^*2*^ = 54.8%; *P* = 0.009); diameter greater than 20 mm (*I*^*2*^ = 90.4%; *P* < 0.001); and location on cheek (*I*^*2*^ = 50.1%; *P* = 0.111), ear (*I*^*2*^ = 39.9%; *P* = 0.092), and temple (*I*^*2*^ = 62.0%; *P* = 0.015), but no significant heterogeneity for Breslow greater than 2 mm (*I*^*2*^ = 30.6%; *P* = 0.156), immunosuppression status (*I*^*2*^ = 33.0%; *P* = 0.176), and location on lip (*I*^*2*^ = 33.5%; *P* = 0.150). The pooled conclusions were stable and not changed by sequential exclusion of individual studies (S2 Appendix). Finally, there was no significant publication bias for the role of poor differentiation (Egger: *P* = 0.475; Begg: *P* = 0.487), perineural invasion (Egger: *P* = 0.104; Begg: *P* = 0.161), Breslow greater than 2 mm (Egger: *P* = 0.696; Begg: *P* = 0.876), and immunosuppression status (Egger: *P* = 0.309; Begg: *P* = 0.764). The potential significant publication bias for diameter greater than 20 mm (Egger: *P* = 0.002; Begg: *P* = 0.640) (S3 Appendix) and the conclusion were not changed after adjustment for potential publication bias using the trim and fill method [63].

### Disease-specific death

**Fig 4:**
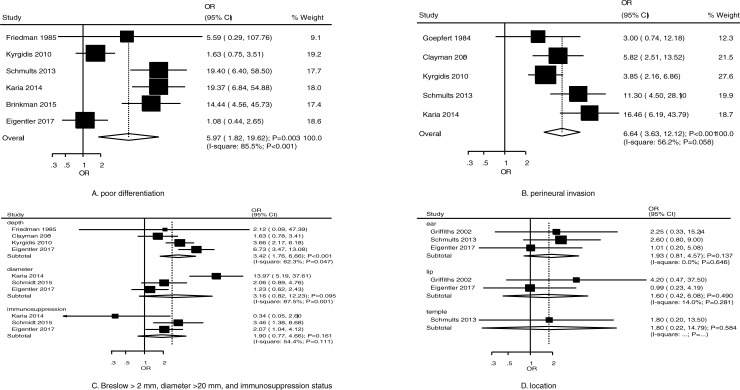
Risk factors for DSD in patients with cSCC.

The summary results for the role of clinicopathological characteristics on the risk of DSD for patients with cSCC are shown in Fig 4. The summary ORs indicated that poor differentiation (OR, 5.97; 95% CI, 1.82–19.62; *P* = 0.003) (Fig 4A), perineural invasion (OR, 6.64; 95% CI, 3.63–12.12; *P* < 0.001) (Fig 4B), and Breslow greater than 2 mm (OR, 3.42; 95% CI, 1.76–6.66; *P* < 0.001) (Fig 4C) were associated with an increased risk of DSD. Diameter greater than 20 mm (OR, 3.16; 95% CI, 0.82–12.23; *P* = 0.095) (Fig 4C), immunosuppression status (OR, 1.90; 95% CI, 0.77–4.66; *P* = 0.161) (Fig 4C), location on ear (OR, 1.93; 95% CI, 0.81–4.57; *P* = 0.137) (Fig 4D), location on lip (OR, 1.60; 95% CI, 0.42–6.08; *P* = 0.490) (Fig 4D), and location on temple (OR, 1.80; 95% CI, 0.22–14.79; *P* = 0.584) (Fig 4D) did not affect the risk of DSD. Moreover, there was potential significant heterogeneity for poor differentiation (*I*^*2*^ = 85.5%; *P* < 0.001), perineural invasion (*I*^*2*^ = 56.2%; *P* = 0.058), Breslow greater than 2 mm (*I*^*2*^ = 62.3%; *P* = 0.047), diameter greater than 20 mm (*I*^*2*^ = 87.5%; *P* < 0.001), and immunosuppression status (*I*^*2*^ = 54.4%; *P* = 0.111), but no significant heterogeneity for location on ear (*I*^*2*^ = 0.0%; *P* = 0.646) or lip (*I*^*2*^ = 14.0%; *P* = 0.281). Furthermore, the pooled conclusions for the role of poor differentiation and perineural invasion on the risk of DSD were robust and not altered by sequential exclusion of individual studies (S2 Appendix). Finally, no significant publication bias was detected for poor differentiation (Egger: *P* = 0.414; Begg: *P* = 1.000) or perineural invasion (Egger: *P* = 0.536; Begg: *P* = 0.462) (S3 Appendix).

## Discussion

The current study was based on observational studies and explored the role of clinicopathological characteristics on the risk of recurrence, metastasis, and DSD. This comprehensive quantitative study included 21,530 patients and 28,627 cSCC from 7 prospective and 36 retrospective studies with a wide range of patient characteristics. The findings of this study found that poor differentiation, perineural invasion, and Breslow greater than 2 mm were associated with an increased risk of recurrence, metastasis, and DSD. Moreover, diameter greater than 20 mm and location on the temple were associated with excess risk of recurrence and metastasis. Finally, location on the ear or lip was associated with an increased risk of metastasis.

A previous systematic review and meta-analysis included 36 studies and reported that the risk of recurrence was significantly increased with Breslow greater than 2 mm, invasion beyond subcutaneous fat, Breslow greater than 6 mm, perineural invasion, and poor differentiation. Invasion beyond subcutaneous fat; Breslow greater than 2 mm; Breslow greater than 6 mm; diameter greater than 20 mm; poor differentiation; perineural invasion; location on temple, ear, and lip; and immunosuppression were associated with an increased risk of metastasis. Furthermore, diameter greater than 20 mm, poor differentiation, location on ear or lip, invasion beyond subcutaneous fat, and perineural invasion were associated with an excess risk of DSD [10]. However, the additional published articles should be entered into a meta-analysis to reevaluate the role of clinicopathological characteristics on the prognosis of cSCC. Therefore, we conducted this systematic review and meta-analysis to provide an accurate predictive value of clinicopathological characteristics on the prognosis of cSCC.

The potential role of clinicopathological characteristics on the risk of recurrence was consistent with that reported in a previous meta-analysis [10]. Nearly all of studies reported similar results or trends. Moreover, most identified prognostic factors for the risk of metastasis were similar to the previous study. However, immunosuppression status was associated with a nonsignificant risk of metastasis, which was not consistent with the previous study [10]. The potential reasons could be accurate follow-up of immunocompromised patients for early diagnosis of cSCC patients in tertiary care centers and that the number of high-risk cSCCs among immunocompromised and immunocompetent patients were not balanced, which could affect the prognosis of cSCC. Moreover, although the role of most disease characteristics on the risk of DSD were consistent with a previous meta-analysis, the role of diameter, location on ear, and location on lip on the risk of DSD were inconsistent with the previous study. The potential reason for this could be that the results of previous study for the role of diameter, location on ear, and location on lip on the risk of DSD were based on only a single study. These results vary, and further large-scale prospective studies are needed for verification.

Several advantages of this study should be highlighted. First, most of the included studies were of relative high quality, and the pooled conclusions were robust. Second, the large sample size allowed us to quantitatively assess the role of clinicopathological characteristics on the prognosis of cSCC. Thus, our findings are potentially more robust than are those of any individual study. Third, the analysis of this study provides comprehensive results regarding the role of differentiation, perineural invasion, depth, diameter, immunosuppression status, and location on the risk of recurrence, metastasis, and DSD.

Despite these advantages, however there are also limitations that should be acknowledged. Most of the included studies were retrospective in design, and selection or recall bias was inevitable. In addition, significant heterogeneity was detected for several of the investigated outcomes, which was not fully interpreted by sensitivity analyses. Third, most of the reported results were unadjusted data, and the results could be affected by the patient characteristics. Fourth, the role of clinicopathological characteristics on the risk of DSD was reported in a smaller number of studies, and the conclusions might vary. Also, publication bias was inevitable because of the analysis based on published articles. Finally, the analysis was based on the study level, and individual data were not available.

## Conclusion

In conclusion, this study indicated that the risk of recurrence, metastasis, and DSD among cSCC patients are significantly increased when patients are characterized by poor differentiation, perineural invasion, and Breslow greater than 2 mm. Moreover, we noted that diameter greater than 20 mm and location on the temple were associated with an increased risk of recurrence and metastasis. Furthermore, location on the ear or lip could result in an excess risk of metastasis in patients with cSCC. The results of this study should be verified by further prospective cohort studies.

## Supporting information

S1 Checklist (DOC)

S1 Appendix Search strategy. (DOCX)

S2 Appendix Sensitivity analysis. (DOCX)

S3 Appendix Funnel plot. (DOCX)
