# Supplementary material for: Identifying risk factors for the prognosis of head and neck cutaneous squamous cell carcinoma: A systematic review and meta-analysis
Source: PLoS One. 2020 Sep 29;15(9):e0239586. doi: 10.1371/journal.pone.0239586 (PMC7523977; doi:10.1371/journal.pone.0239586)
Supplement: S1 Appendix — (DOCX) [file pone.0239586.s002.docx]

1. exp *Carcinoma, Squamous Cell/
2. exp *Skin Neoplasms/
3. 1 and 2
4. (skin or cutaneous or dermal or cutanea).ti.
5. 1 and 4
6. from 3 keep 40382-48337
7. ((skin or cutaneous or dermal or cutanea) adj3 ((squamous or epidermoid or planocellular or "prickle cell" or verrucous) adj2 carcinoma*)).ti,ab.
8. 5 or 6 or 7
9. exp Risk/
10. exp Risk Factors/
11. exp Neoplasm Invasiveness/
12. exp Nervous System/
13. 11 and 12
14. exp perineural invasion/
15. exp tumor volume/
16. exp Tumor Burden/
17. exp Ear Neoplasms/ or exp Ear/
18. exp Cheek/
19. exp Lip/ or exp Lip Neoplasms/
20. exp lip carcinoma/
21. exp tumor differentiation/
22. exp Immunocompromised Host/
23. exp immunocompromised patient/
24. exp sentinel lymph node/ 15314
25. exp sentinel lymph node metastasis/
26. exp Sentinel Lymph Node Biopsy/
27. (risk or risks or "perineural invasion*" or "peri-neural invasion*" or PNI or depth or thickness or size or diameter or ((tumor or tumour or neoplasm*) adj (burden or load or weight* or volume)) or location* or ear or ears or cheek* or lip or lips or differentiation or immunocompromised or "immune compromi*" or "sentinel lymph node*").ti,ab.
28. or/13-27
29. 9 or 10 or 28
30. exp "outcome assessment (health care)"/
31. exp "outcome and process assessment (health care)"/
32. exp Mortality/
33. mo.fs.
34. exp Treatment Outcome/
35. exp Disease-Free Survival/ or exp Survival Analysis/ or exp Survival/ or exp Survival Rate/
36. exp Death/
37. exp Morbidity/
38. exp cancer control/
39. exp Recurrence/ or exp Neoplasm Recurrence, Local/
40. exp recurrent disease/
41. exp cancer recurrence/
42. exp Neoplasm Metastasis/
43. (outcome* or recurrence* or relaps* or recrudescence* or recurrent or recidive or metastas* or metastatic* or spread* or disseminat* or secondary or migrat* or death or morbidity or mortality or surviv*).ti,ab.
44. sc.fs.
45. from 44 keep 109911-242775
46. or/30-43
47. 45 or 46
48. 8 and 29 and 47
49. exp evidence based medicine/
50. exp meta analysis/
51. exp Meta-Analysis as Topic/
52. exp "systematic review"/
53. exp controlled study/
54. exp Randomized Controlled Trial/
55. exp triple blind procedure/
56. exp Double-Blind Method/
57. exp Single-Blind Method/
58. exp latin square design/
59. exp Placebos/
60. exp Placebo Effect/
61. exp comparative study/
62. exp Cross-Sectional Studies/
63. exp Cross-Over Studies/
64. exp Cohort Studies/
65. exp longitudinal study/
66. exp retrospective study/
67. exp prospective study/
68. exp population research/
69. exp observational study/
70. exp clinical trial/
71. clinical study/
72. exp Evaluation Studies/
73. exp Evaluation Studies as Topic/
74. exp quantitative study/
75. exp validation studies/
76. in vivo study/
77. exp panel study/
78. exp Pilot Projects/
79. exp pilot study/
80. exp prevention study/
81. exp replication study/
82. exp Feasibility Studies/
83. exp trend study/
84. exp correlational study/
85. exp case-control studies/
86. exp confidence interval/
87. exp regression analysis/
88. exp proportional hazards model/
89. exp multivariate analysis/
90. "limit follow up studies to medline only. embase maps to follow up".ti.
91. exp follow up studies/
92. exp case study/
93. "limit case study above to embase only. medline maps to case report".ti.
94. odds ratio/
95. "limit odds ratio above to embase. medline maps to risk".ti.
96. ((evidence adj based) or (meta adj analys*) or (systematic* adj3 review*) or (control* adj2 study) or (control* adj2 trial) or (randomized adj2 study) or (randomized adj2 trial) or (randomised adj2 study) or (randomised adj2 trial) or (doubl* adj blind*) or (doubl* adj mask*) or (singl* adj blind*) or (singl* adj mask*) or (tripl* adj blind*) or (tripl* adj mask*) or (trebl* adj blind*) or (trebl* adj mask*) or "latin square" or placebo* or multivariate or "comparative study" or "comparative survey" or "comparative analysis" or (intervention* adj2 study) or (intervention* adj2 trial) or "cross-sectional study" or "cross-sectional analys*" or "cross-sectional survey*" or "crosssectional design*" or "prevalence study" or "prevalence analys*" or "prevalence survey*" or "disease frequency study" or "disease frequency analys*" or "disease frequency survey*" or crossover or "cross-over" or "cohort study" or "cohort survey" or "cohort analysis" or "longitudinal study" or "longitudinal survey" or "longitudinal analysis" or "retrospective study" or "retrospective survey" or "retrospective analysis" or "prospective study" or "prospective survey" or "prospective analysis" or "population study" or "population survey" or "population analysis" or "concurrent study" or "concurrent survey" or "concurrent analysis" or "incidence study" or "incidence survey" or "incidence analysis" or "follow-up study" or "follow-up survey" or "follow-up analysis" or "observational study" or "observational survey" or "observational analysis" or "case study" or "case series" or "clinical series" or "case studies" or "clinical study" or "clinical trial" or "evaluation study" or "evaluation survey" or "evaluation analysis" or "quantitative study" or "quantitative analys*" or "validation study" or "validation survey" or "validation analysis" or "in vivo study" or "in vivo analysis" or "panel study" or "panel survey" or "panel analysis" or "pilot study" or "pilot survey" or "pilot analysis" or "prevention study" or "prevention survey" or "prevention analysis" or "replication study" or "replication analysis" or "feasibility study" or "feasibility analysis" or "trend study" or "trend survey" or "trend analysis" or (correlaton* adj study) or (correlaton* adj analys*) or "case control study" or "case base study" or "case referrent study" or "case referent study" or "case compeer study" or "case comparison study" or "odds ratio" or "confidence interval" or cohort* or "regression analysis" or "hazards model*" or "multivariate analysis" or "change analysis").ti,ab.
97. from 91 keep 871715-1389013
98. from 92 keep 1-29348
99. from 94 keep 1-370946
100. or/49-89
101. or/96-100
102. 48 and 101
103. from 48 keep 1206-2884
104. limit 103 to (clinical trial, all or clinical trial, phase i or clinical trial, phase ii or clinical trial, phase iii or clinical trial, phase iv or clinical trial or comparative study or controlled clinical trial or meta analysis or multicenter study or observational study or pragmatic clinical trial or randomized controlled trial or systematic reviews or validation studies)
105. 102 or 104
106. limit 105 to (book or book series or editorial or erratum or letter or note or addresses or autobiography or bibliography or biography or comment or dictionary or directory or interactive tutorial or interview or lectures or legal cases or legislation or news or newspaper article or overall or patient education handout or periodical index or portraits or published erratum or video-audio media or webcasts)
107. 105 not 106
108. from 48 keep 2885-2907
109. 107 not (exp animals/ not exp humans/)
110. 108 or 109
111. remove duplicates from 110
