# Supplementary material for: Identifying risk factors for the prognosis of head and neck cutaneous squamous cell carcinoma: A systematic review and meta-analysis
Source: PLoS One. 2020 Sep 29;15(9):e0239586. doi: 10.1371/journal.pone.0239586 (PMC7523977; doi:10.1371/journal.pone.0239586)
Supplement: S2 Appendix — (DOCX) [file pone.0239586.s003.docx]

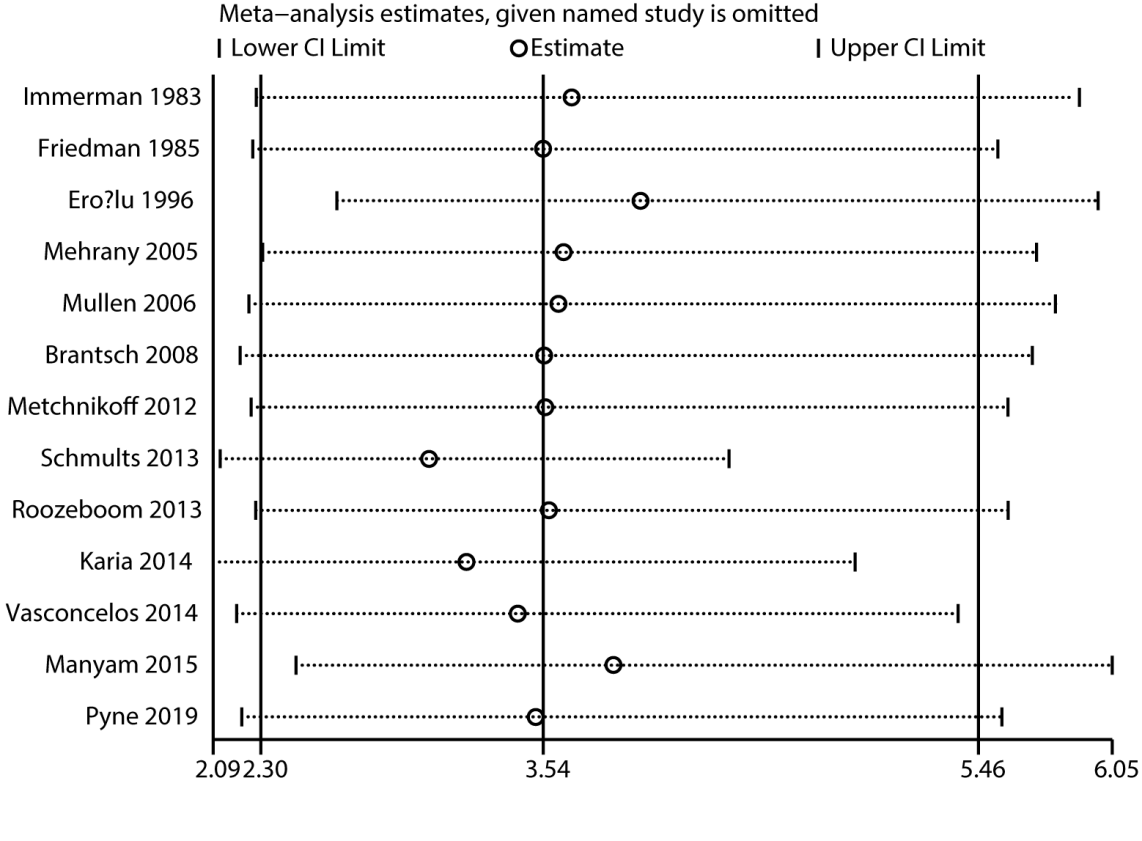


Figure S1. Sensitivity analysis for the role of poor differentiation on the risk of recurrence in patients with cSCC.


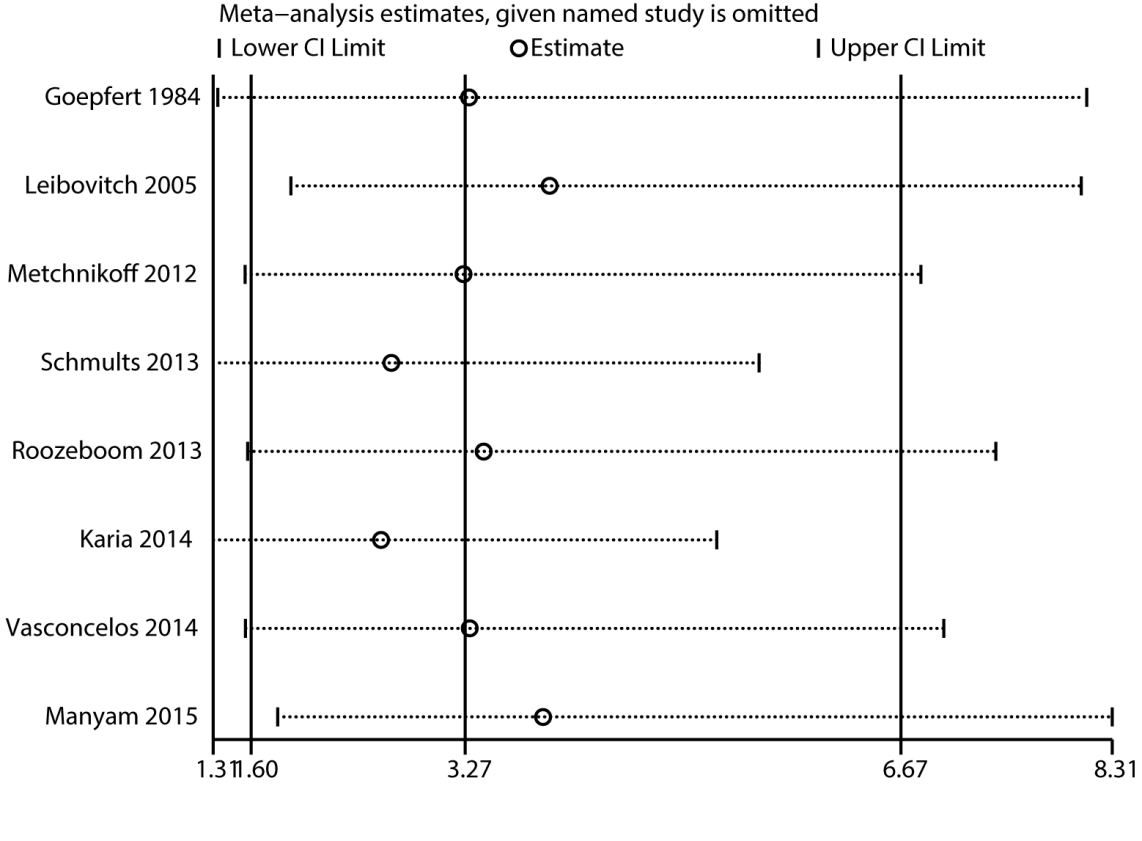


Figure S2. Sensitivity analysis for the role of perineural invasion on the risk of recurrence in patients with cSCC.


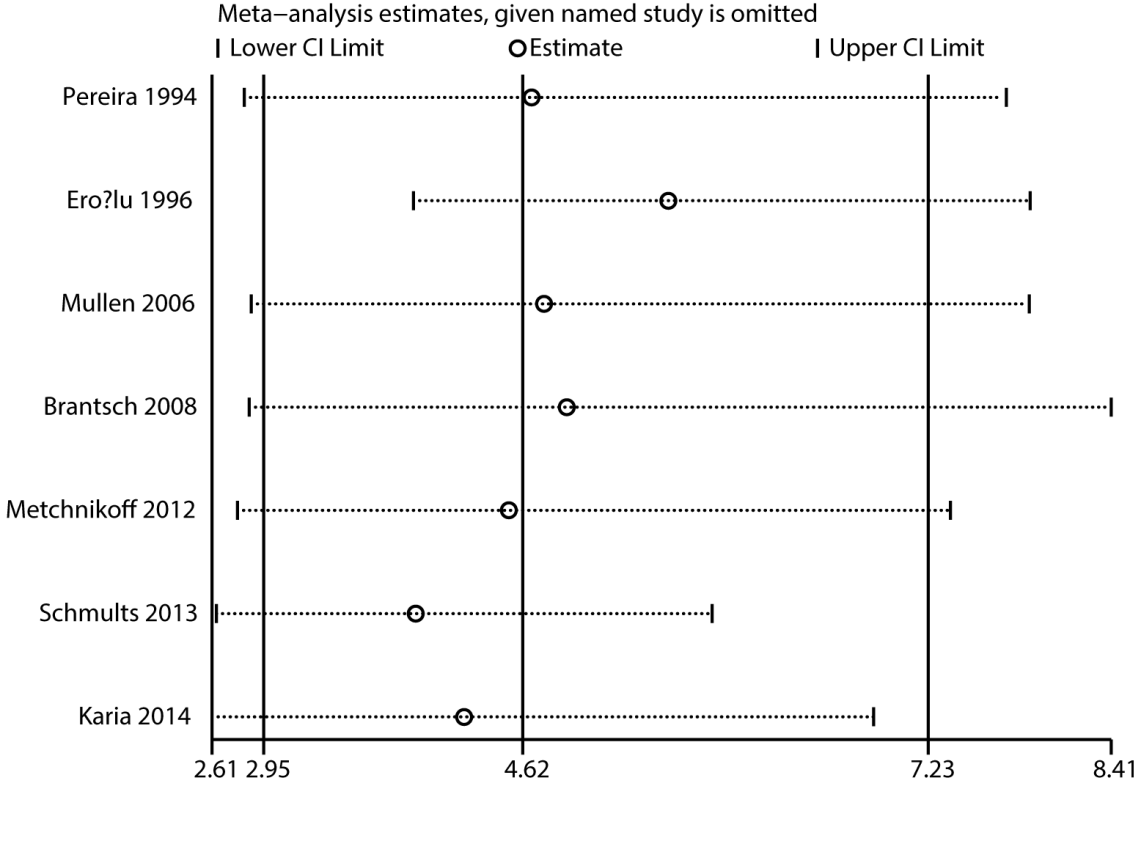


Figure S3. Sensitivity analysis for the role of diameter >20 mm on the risk of recurrence in patients with cSCC.


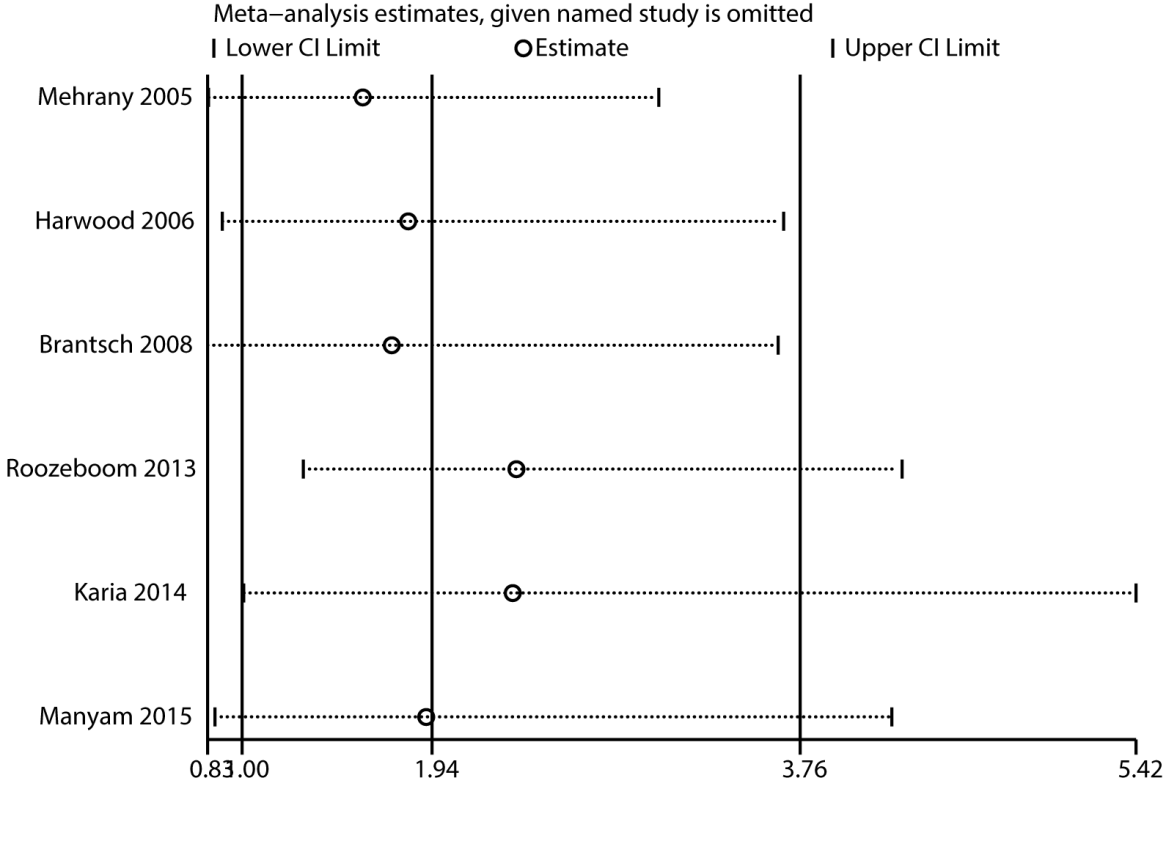


Figure S4. Sensitivity analysis for the role of immunosuppression status on the risk of recurrence in patients with cSCC.


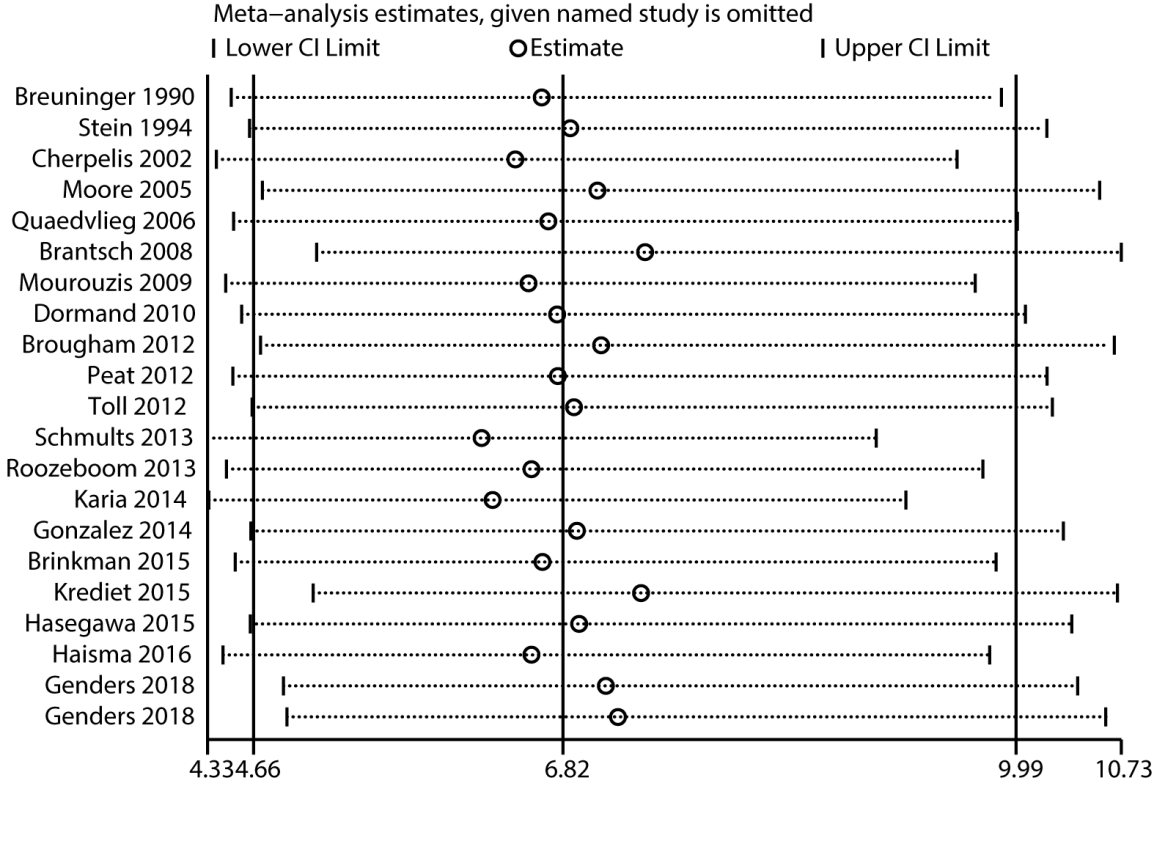


Figure S5. Sensitivity analysis for the role of poor differentiation on the risk of metastasis in patients with cSCC.


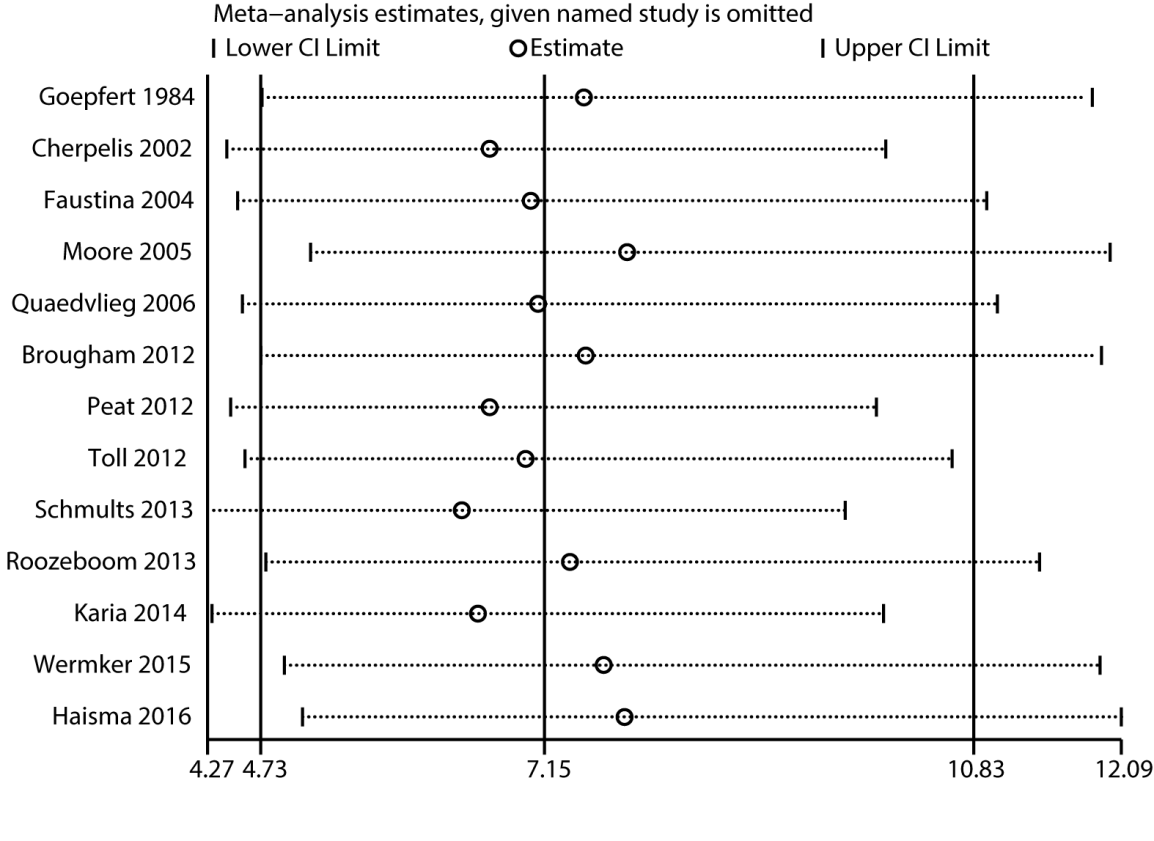


Figure S6. Sensitivity analysis for the role of perineural invasion on the risk of metastasis in patients with cSCC.


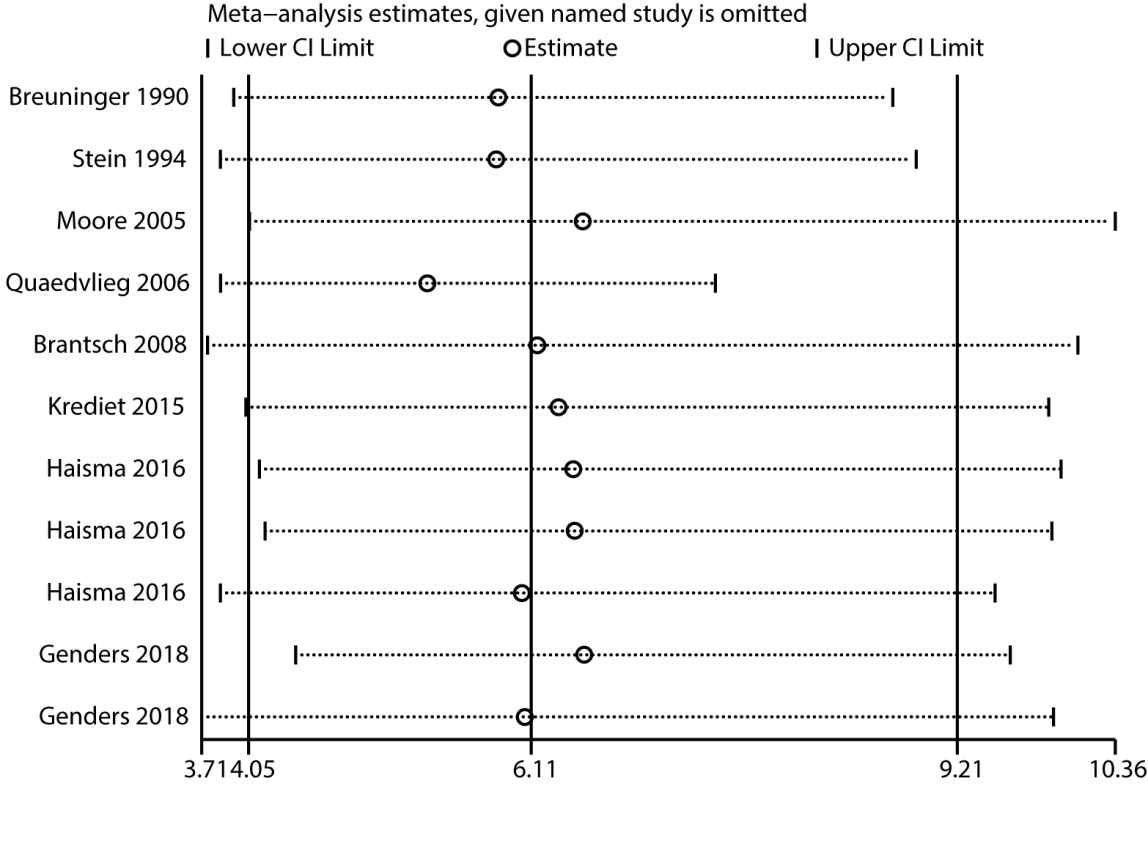


Figure S7. Sensitivity analysis for the role of Breslow > 2 mm on the risk of metastasis in patients with cSCC.


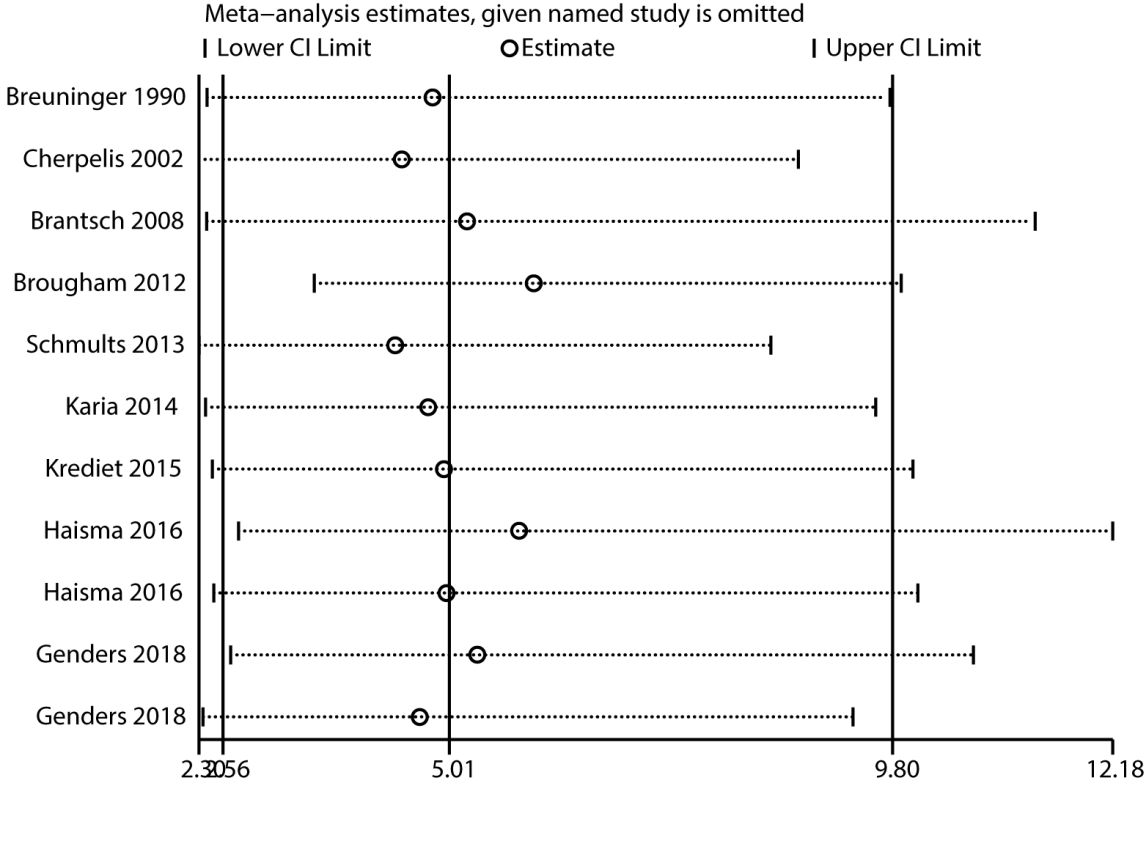


Figure S8. Sensitivity analysis for the role of diameter >20 mm on the risk of metastasis in patients with cSCC.


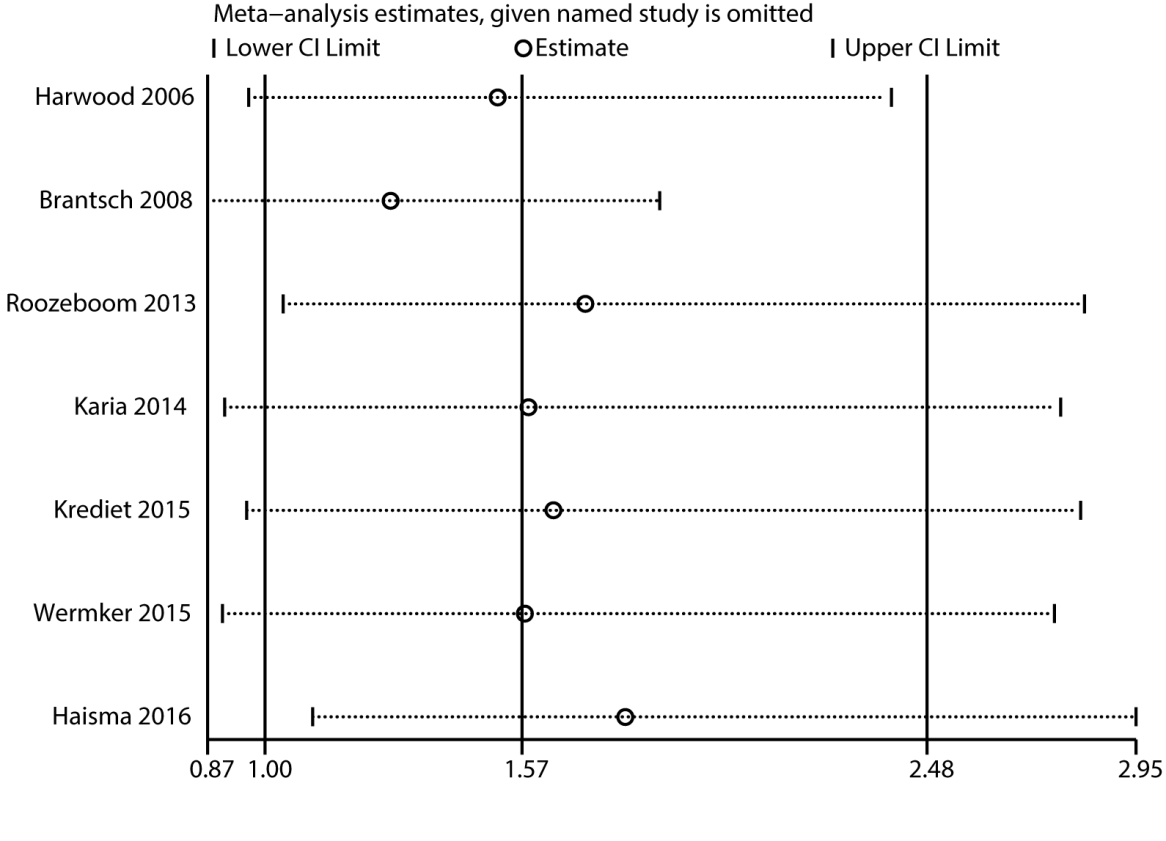


Figure S9. Sensitivity analysis for the role of immunosuppression status on the risk of metastasis in patients with cSCC.


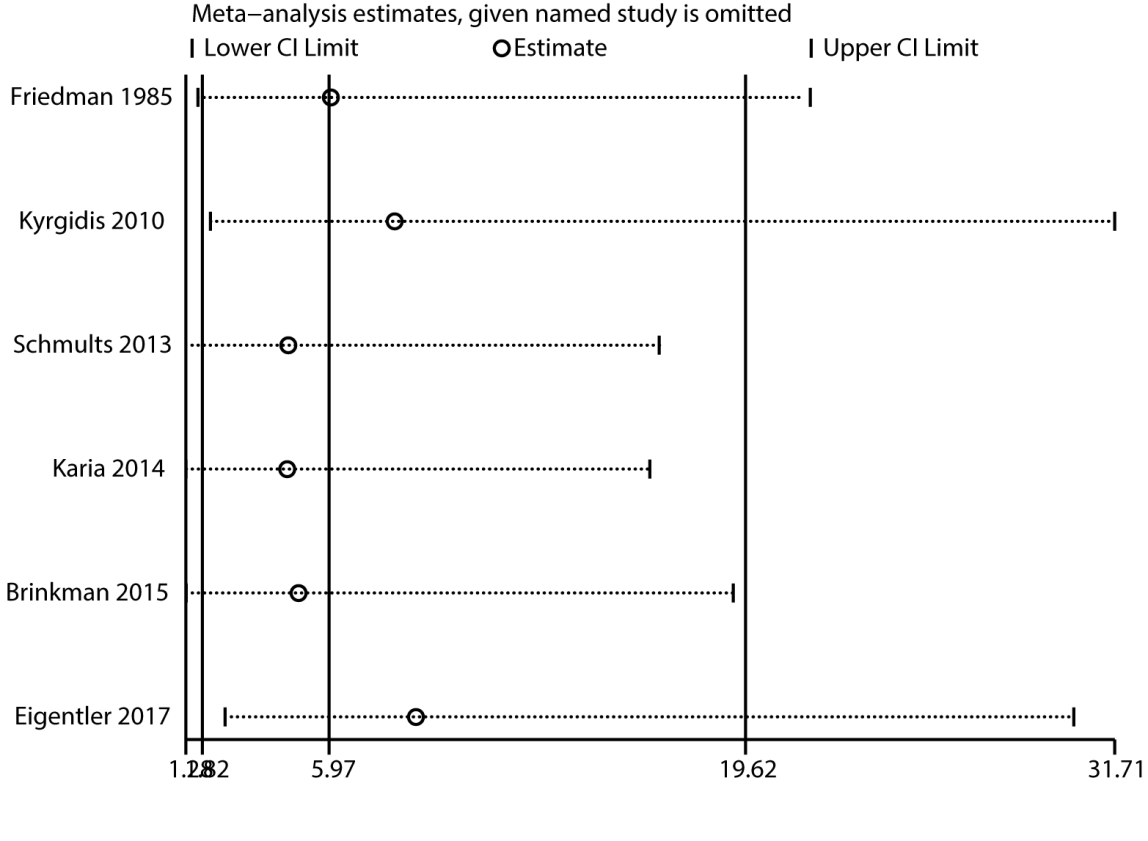


Figure S10. Sensitivity analysis for the role of poor differentiation on the risk of DSD in patients with cSCC.


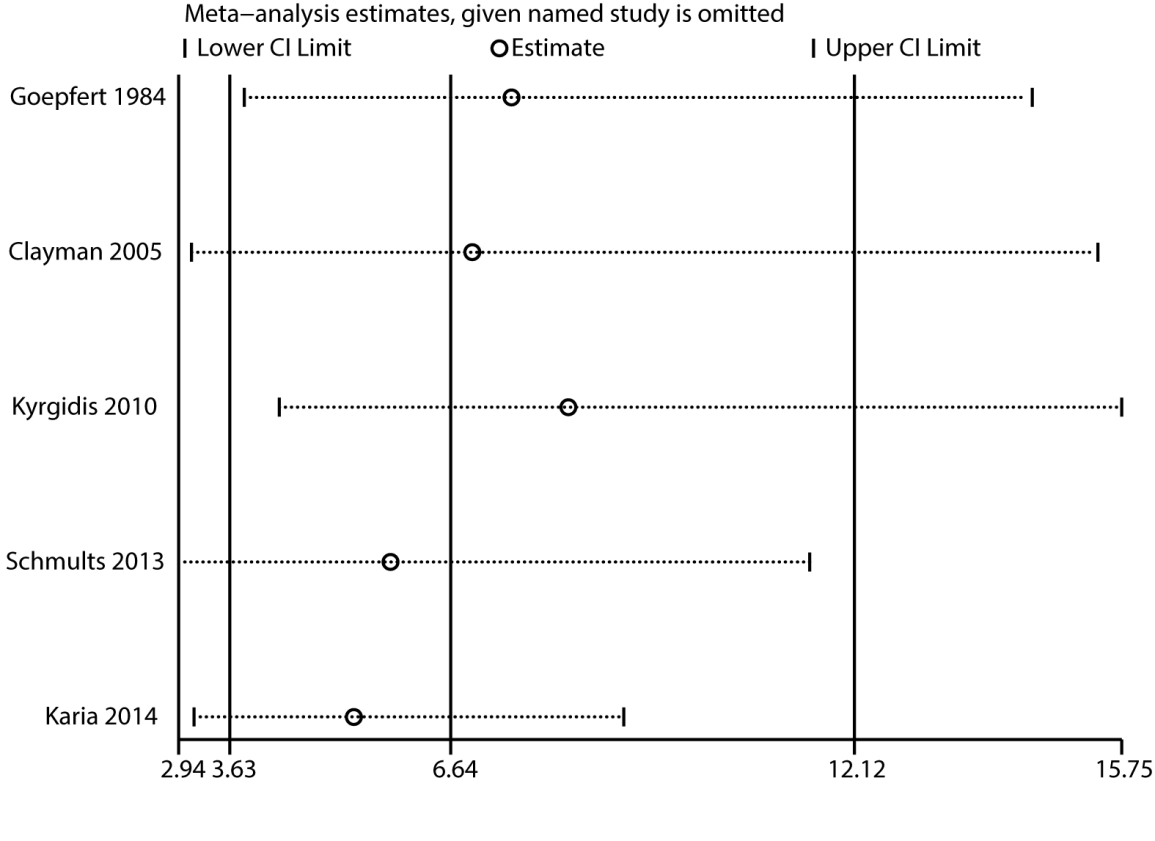


Figure S11. Sensitivity analysis for the role of perineural invasion on the risk of DSD in patients with cSCC.
