# Supplementary material for: Identifying risk factors for the prognosis of head and neck cutaneous squamous cell carcinoma: A systematic review and meta-analysis
Source: PLoS One. 2020 Sep 29;15(9):e0239586. doi: 10.1371/journal.pone.0239586 (PMC7523977; doi:10.1371/journal.pone.0239586)
Supplement: S3 Appendix — (DOCX) [file pone.0239586.s004.docx]

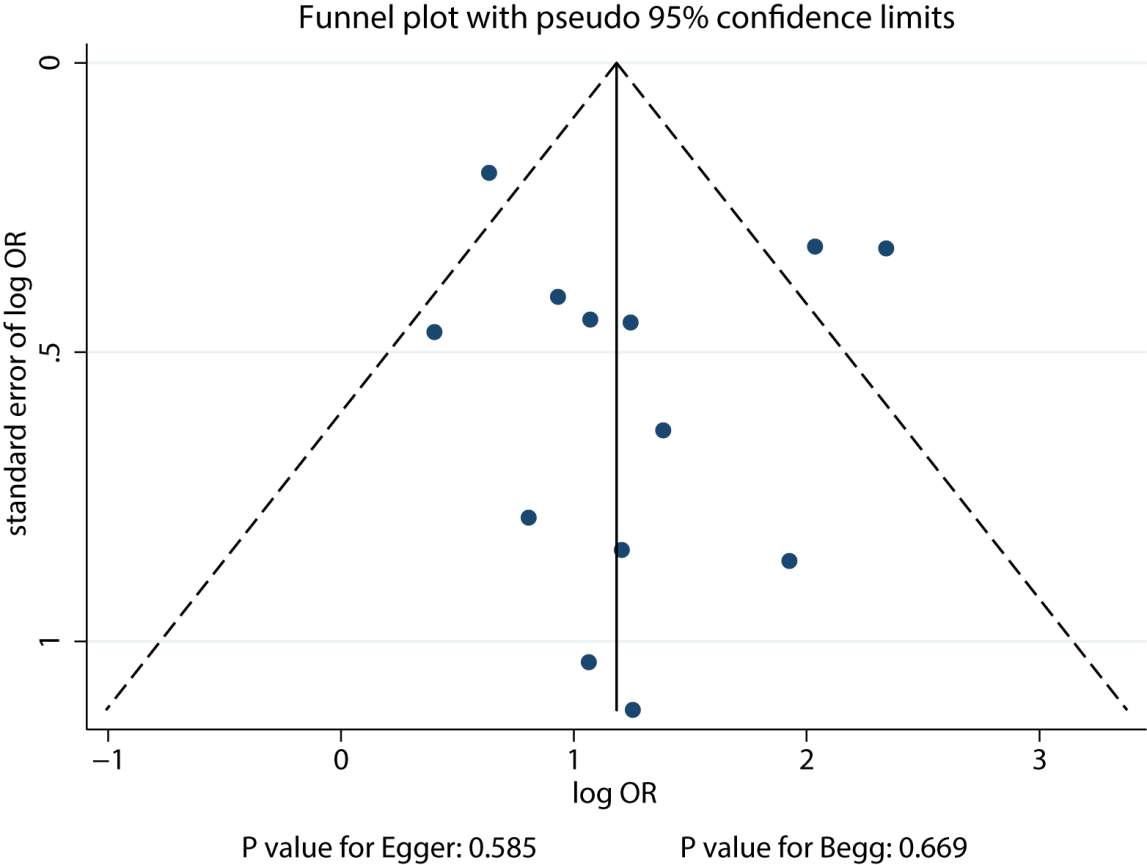


Figure S1. Funnel plot for the role of poor differentiation on the risk of recurrence in patients with cSCC.


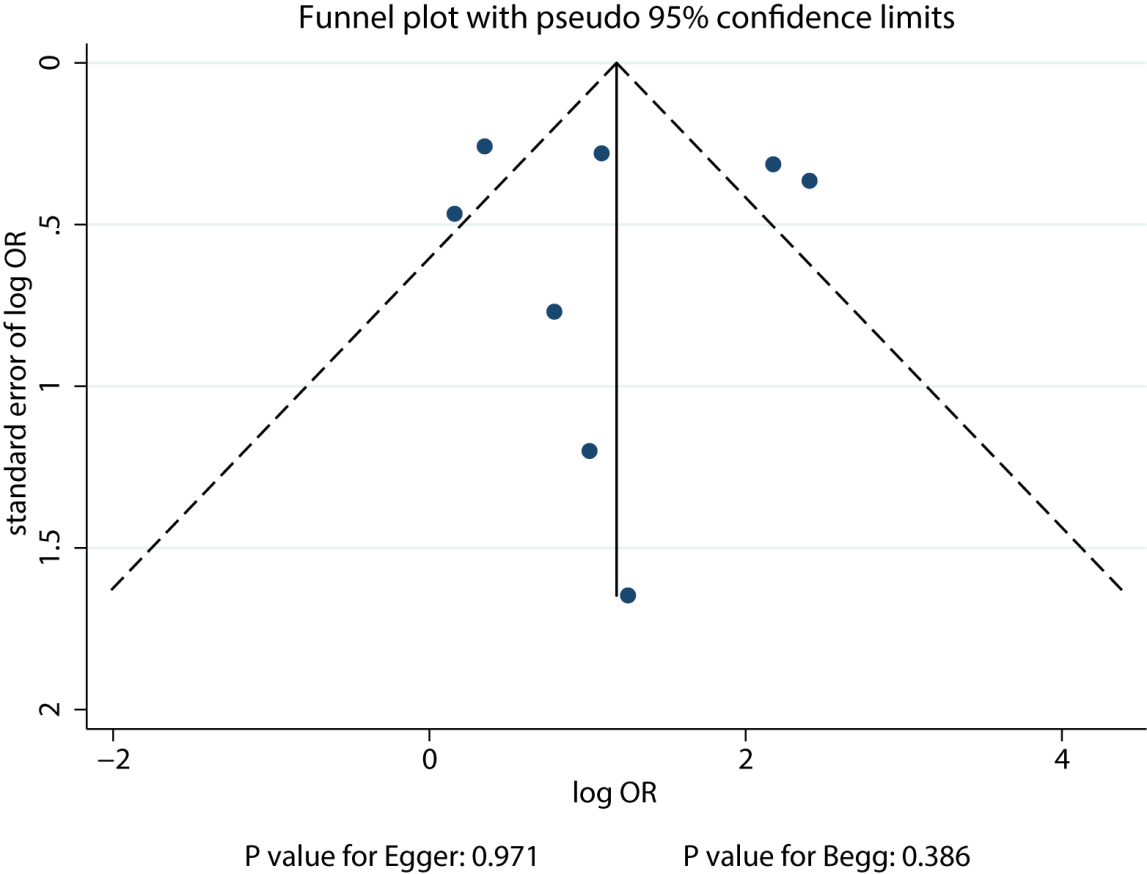


Figure S2. Funnel plot for the role of perineural invasion on the risk of recurrence in patients with cSCC.


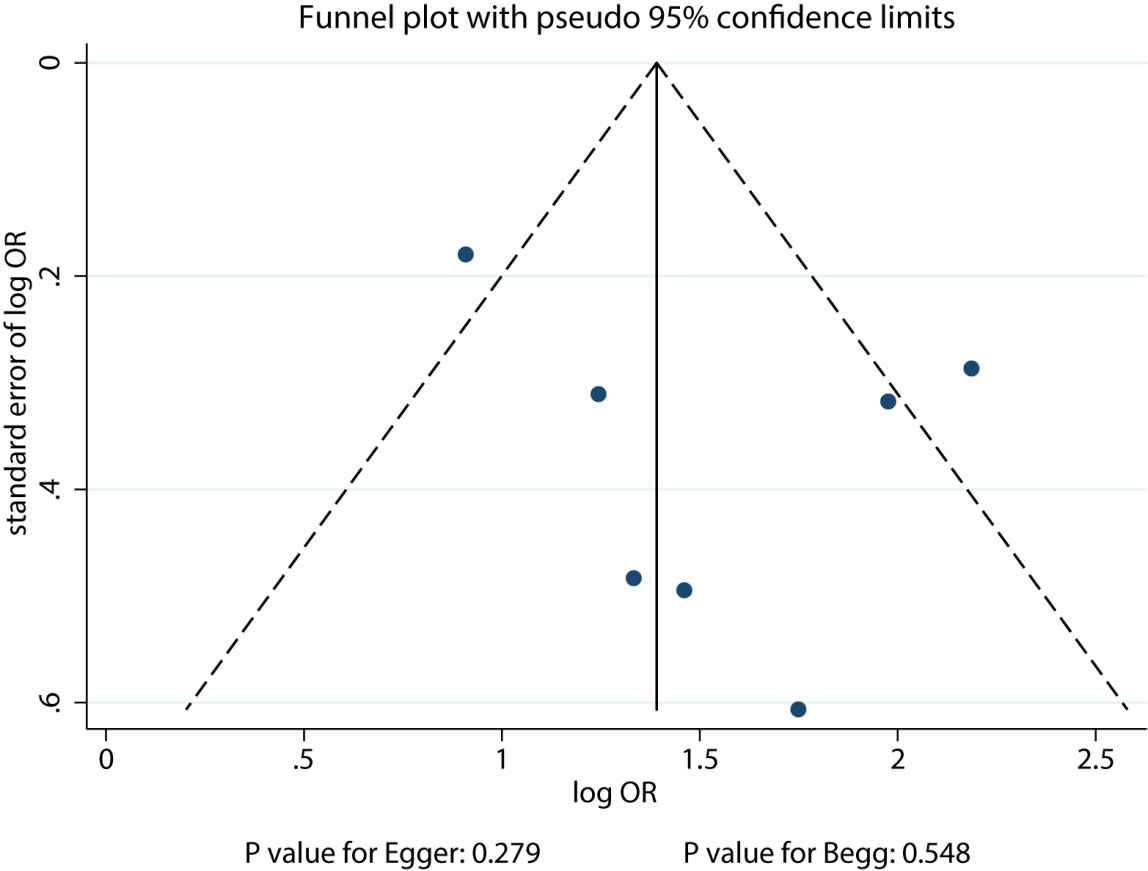


Figure S3. Funnel plot for the role of diameter >20 mm on the risk of recurrence in patients with cSCC.


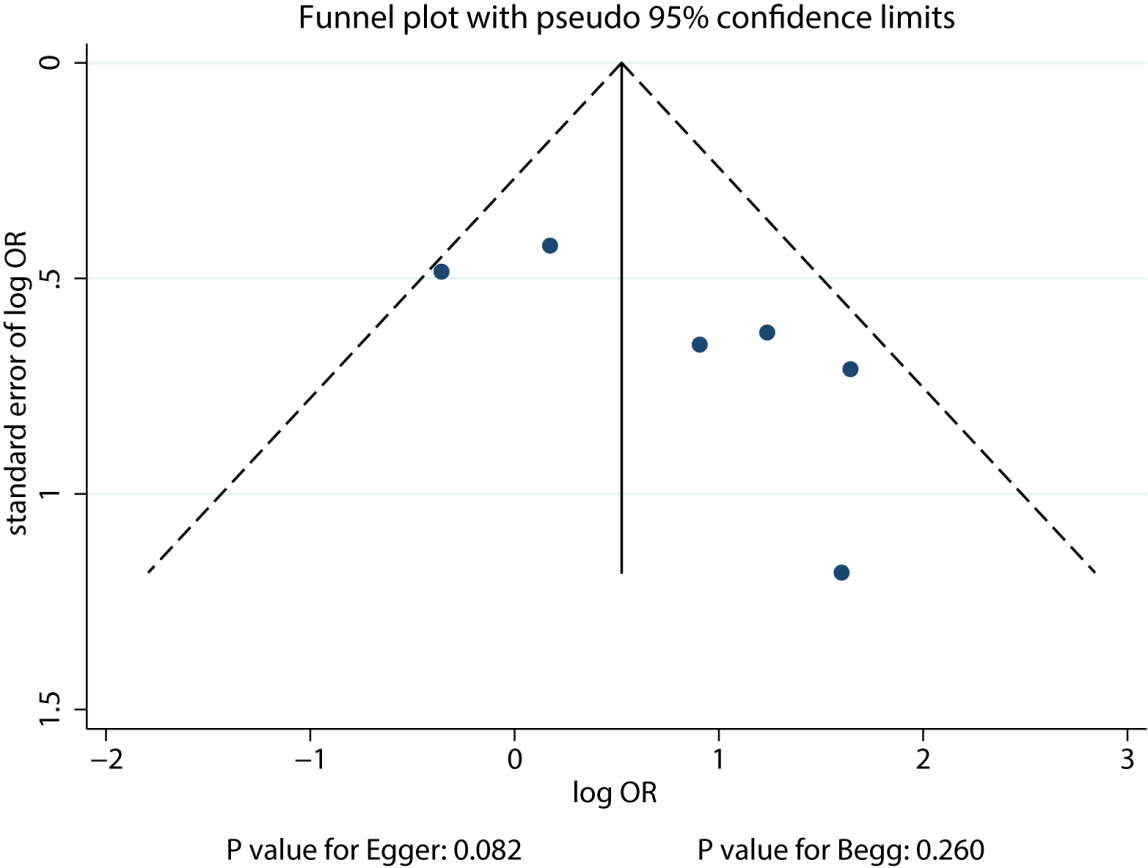


Figure S4. Funnel plot for the role of immunosuppression status on the risk of recurrence in patients with cSCC.


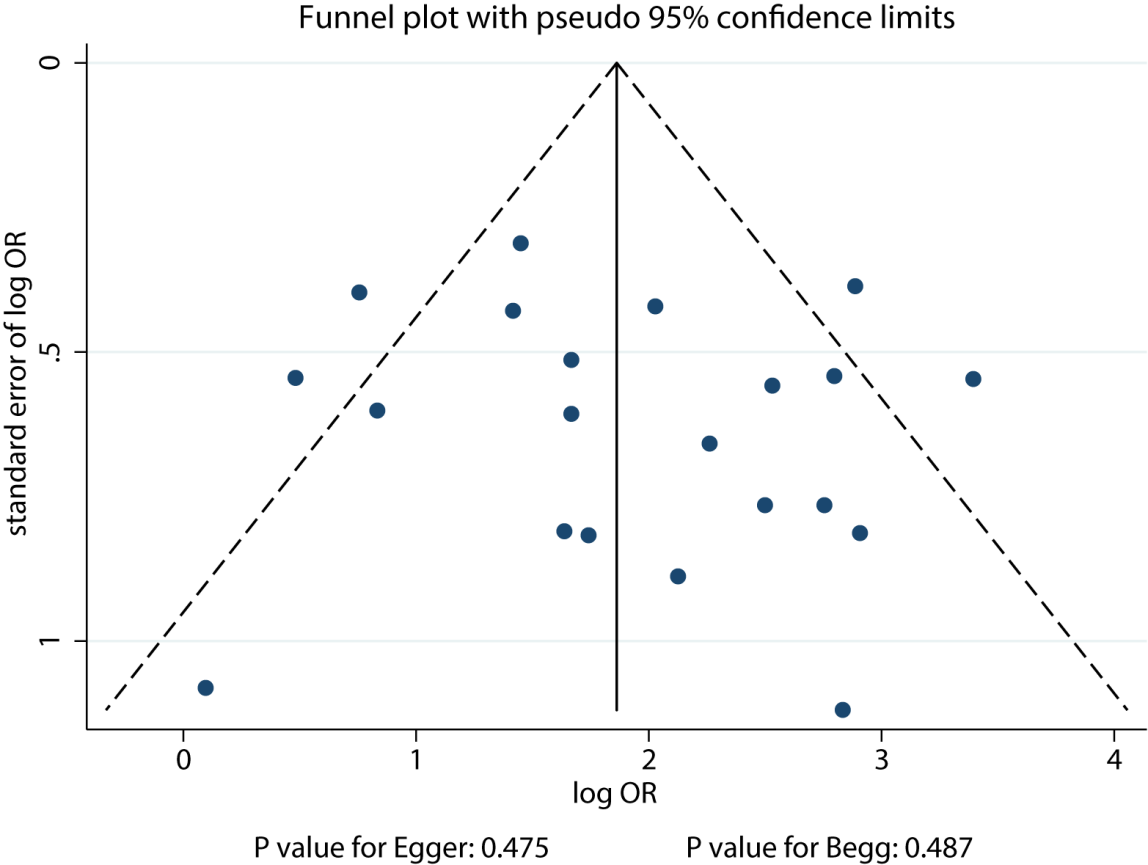


Figure S5. Funnel plot for the role of poor differentiation on the risk of metastasis in patients with cSCC.


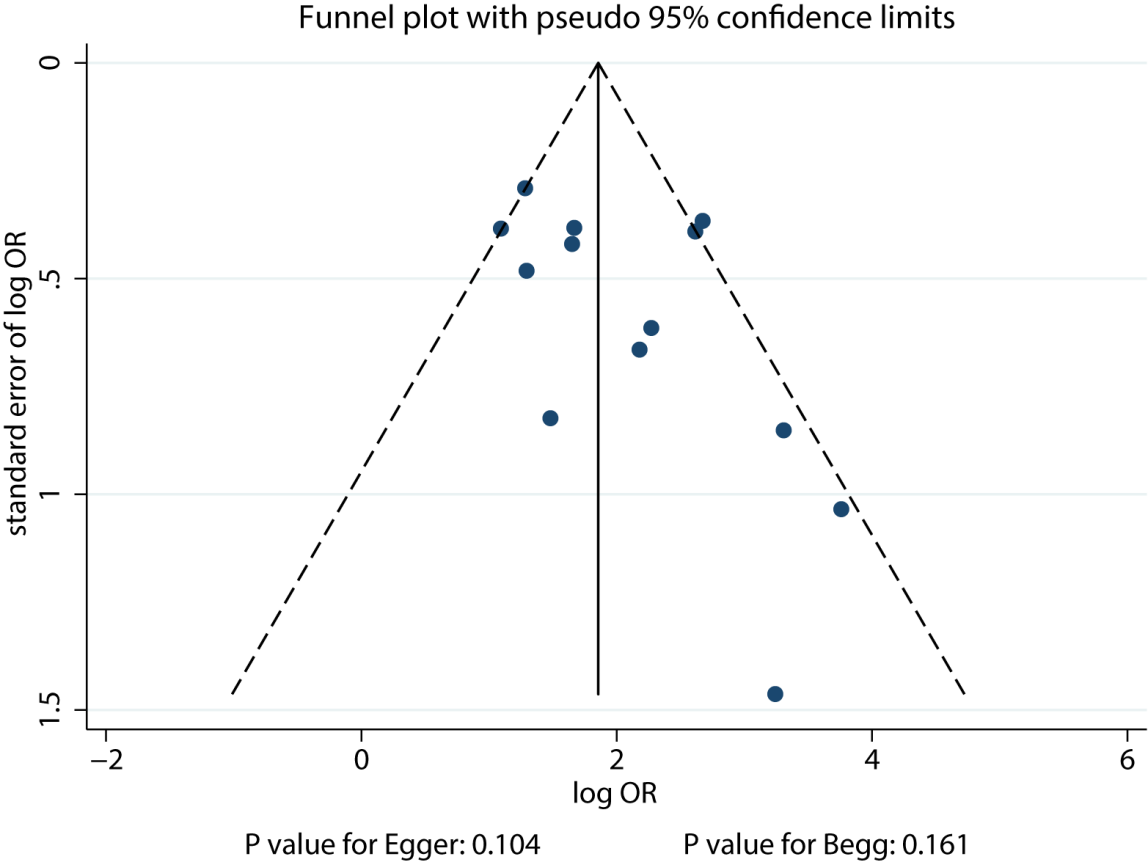


Figure S6. Funnel plot for the role of perineural invasion on the risk of metastasis in patients with cSCC.


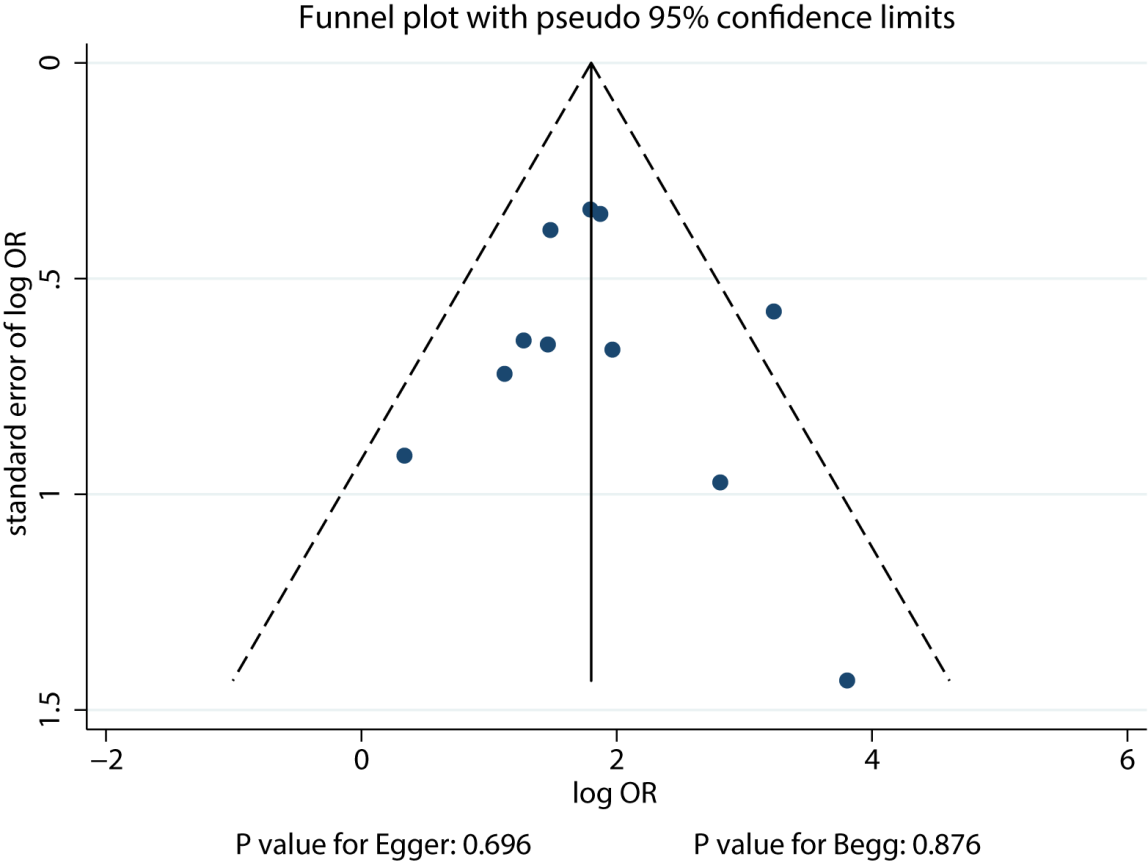


Figure S7. Funnel plot for the role of Breslow > 2 mm on the risk of metastasis in patients with cSCC.


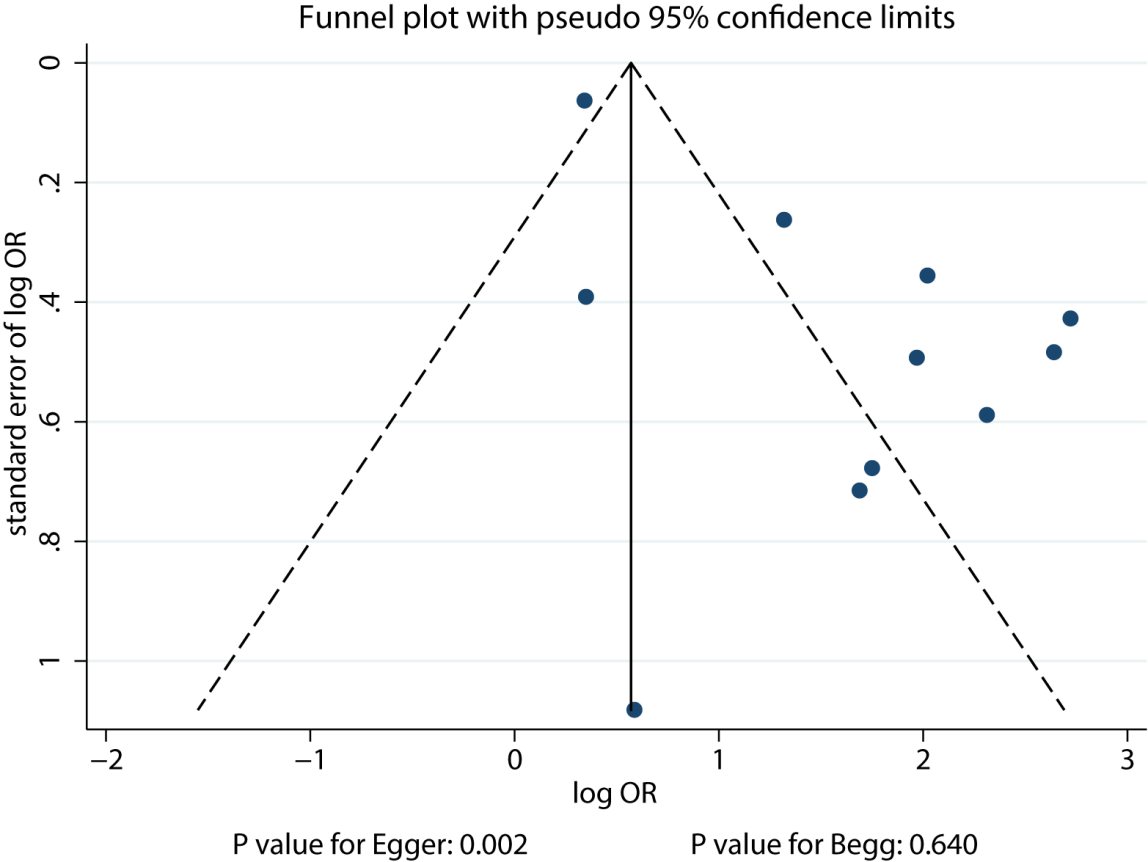


Figure S8. Funnel plot for the role of diameter >20 mm on the risk of metastasis in patients with cSCC.


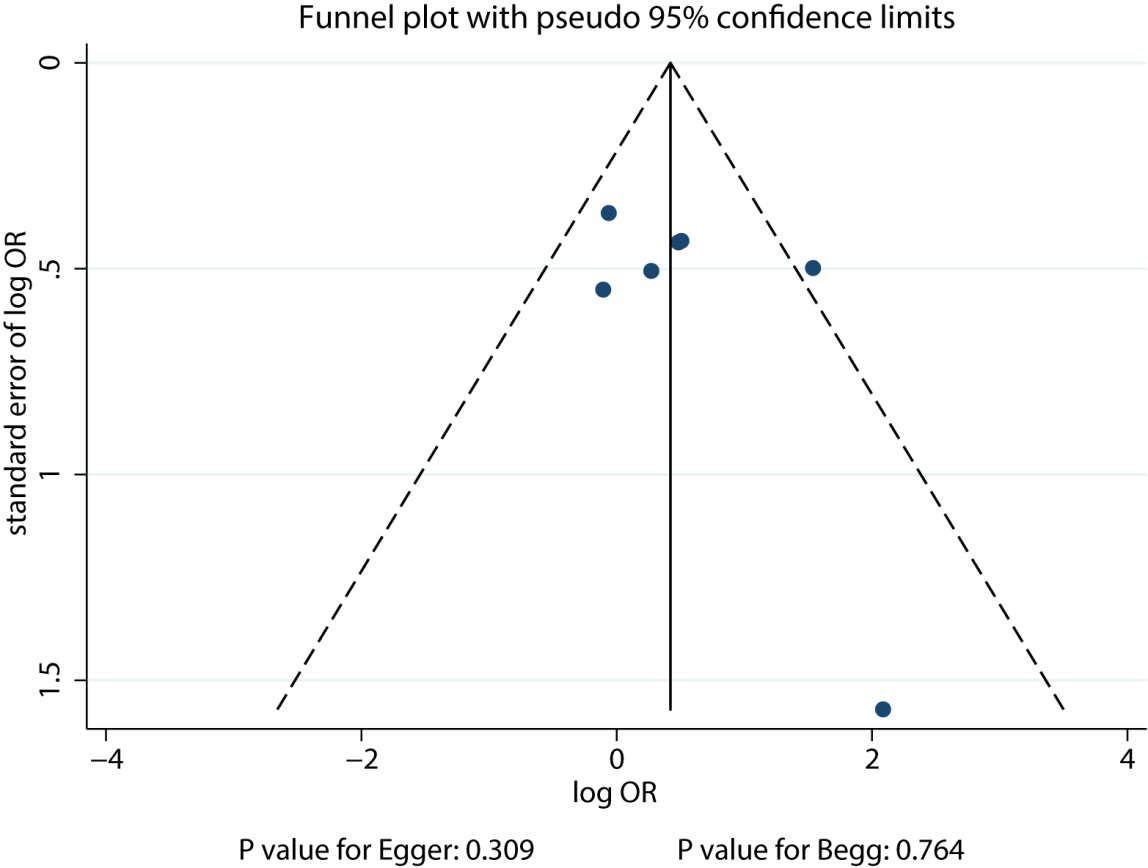


Figure S9. Funnel plot for the role of immunosuppression status on the risk of metastasis in patients with cSCC.


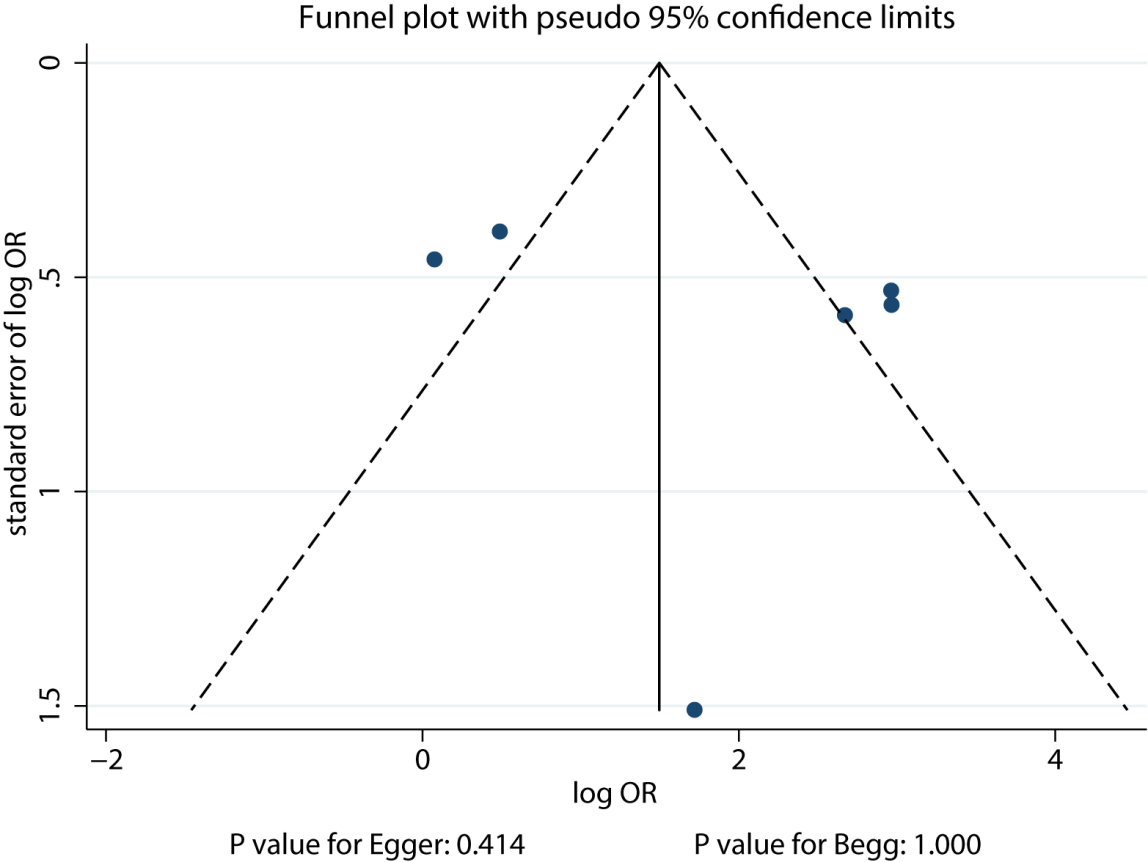


Figure S10. Funnel plot for the role of poor differentiation on the risk of DSD in patients with cSCC.


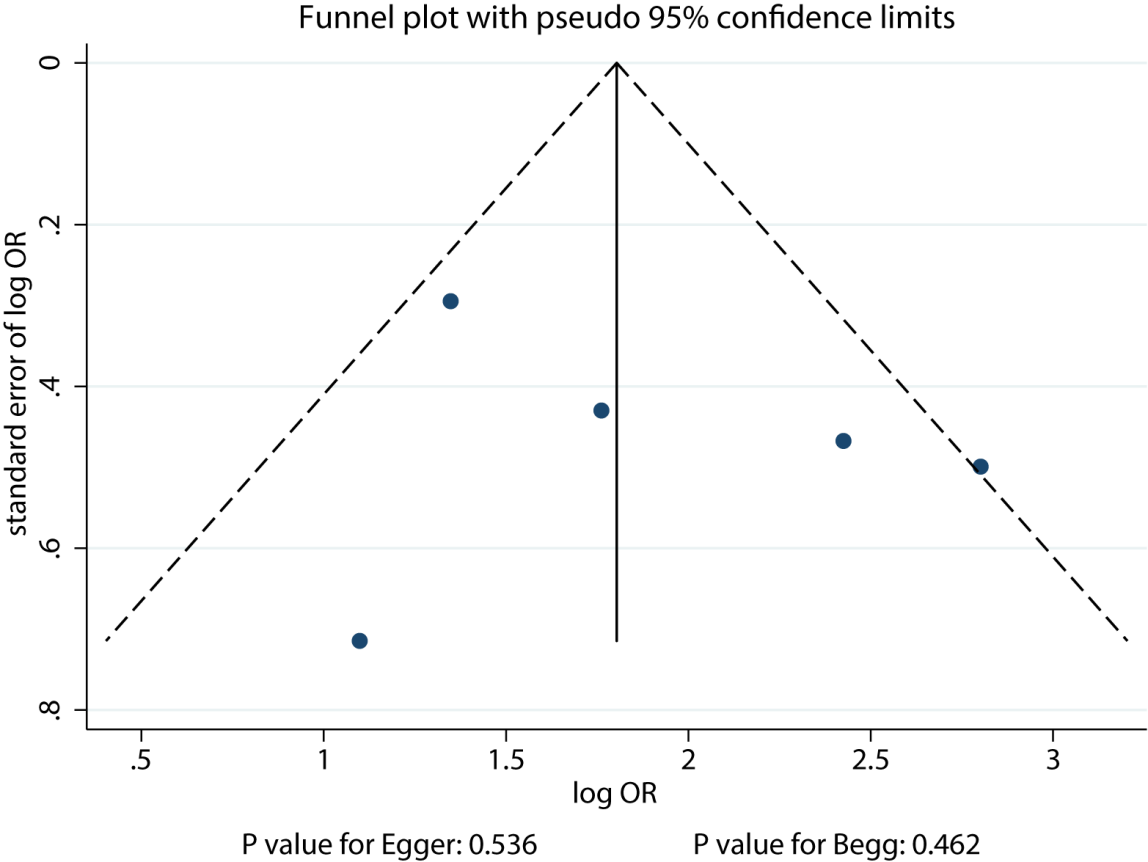


Figure S11. Funnel plot for the role of perineural invasion on the risk of DSD in patients with cSCC.
